# Mitochondrial F0F1-ATP synthase governs the induction of mitochondrial fission

**DOI:** 10.1016/j.isci.2024.109808

**Published:** 2024-04-24

**Authors:** Charlène Lhuissier, Valérie Desquiret-Dumas, Anaïs Girona, Jennifer Alban, Justine Faure, Julien Cassereau, Philippe Codron, Guy Lenaers, Olivier R. Baris, Naïg Gueguen, Arnaud Chevrollier

**Affiliations:** 1 University Angers, MitoLab Team, MitoVasc Unit, CNRS UMR6015, INSERM U1083, SFR ICAT, Angers, France; 2 Departments of Biochemistry and Molecular Biology, University Hospital Angers, Angers, France; 3 Department of Neurology, Angers University Hospital, Angers, France

**Keywords:** Biochemistry, Cell biology, Functional aspects of cell biology

## Abstract

Mitochondrial dynamics is a process that balances fusion and fission events, the latter providing a mechanism for segregating dysfunctional mitochondria. Fission is controlled by the mitochondrial membrane potential (ΔΨm), optic atrophy 1 (OPA1) cleavage, and DRP1 recruitment. It is thought that this process is closely linked to the activity of the mitochondrial respiratory chain (MRC). However, we report here that MRC inhibition does not decrease ΔΨm nor increase fission, as evidenced by hyperconnected mitochondria. Conversely, blocking F0F1-ATP synthase activity induces fragmentation. We show that the F0F1-ATP synthase is sensing the inhibition of MRC activity by immediately promoting its reverse mode of action to hydrolyze matrix ATP and restoring ΔΨm, thus preventing fission. While this reverse mode is expected to be inhibited by the ATPase inhibitor ATPIF1, we show that this sensing is independent of this factor. We have unraveled an unexpected role of F0F1-ATP synthase in controlling the induction of fission by sensing and maintaining ΔΨm.

## Highlights

• Defects in the respiratory chain do not lead to fragmentation of mitochondria
• ATP hydrolysis by F0F1-ATP synthase ensures connectivity of mitochondrial network
• Mitochondrial fission resistance relies on glycolytic energetic metabolism
• F0F1-ATP synthase mutants are prone to MRC-induced mitochondrial fission

## Graphical abstract

**Figure:**
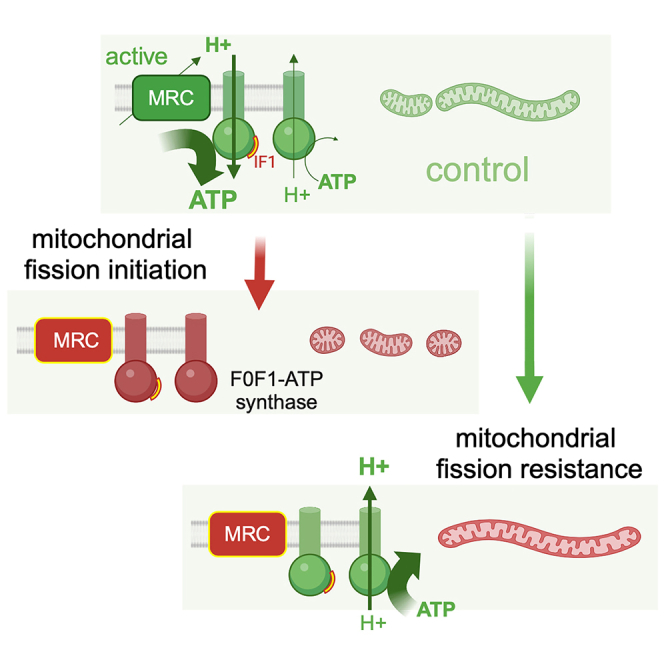

## Introduction

Mitochondria are dynamic organelles organized in tubular networks distributed throughout the cytoplasm. A fine balance between mitochondrial fusion and fission events is important for proper distribution of the organelles, the maintenance of mitochondrial genome, and metabolism. Mitochondrial dynamics is also involved in quality control mechanisms, as dysfunctional mitochondria are isolated from the network to facilitate their degradation by mitophagy.^1^^,^^2^ The dynamin like GTPases DRP1, mitofusin 1 and 2 (MFN1/2), and optic atrophy 1 (OPA1) are the main effectors of this process. Cytosolic DRP1 is recruited to the outer membrane where it promotes mitochondrial tubule constriction and fission by squeezing mitochondrial membranes.^3^ MFN1 and MFN2 induce the fusion of adjacent mitochondrial outer membranes (MOMs),^4^ while the fusion of the mitochondrial inner membranes (MIMs) requires OPA1.^5^ Proteolytic processing of OPA1 contributes to the complex regulation of MIM organization by shaping mitochondrial cristae junction and cristae remodeling.^6^ The long OPA1 isoforms (L-OPA1) and the short ones (S-OPA1) coexist, and their equilibrium is regulated by the action of MIM proteases YME1L and OMA1.^7^ L-OPA1 isoforms, bound to the MIM, are essential for its fusion, while the S-OPA1 isoforms are proposed to limit fusion and promote fission by disrupting cristae structures.^8^^,^^9^ The role of S-OPA1 isoforms in mitochondrial fragmentation is promoted by OPA1 cleavage when OMA1 is over-activated following a decrease of the mitochondrial membrane potential (ΔΨm).^10^^,^^11^ ΔΨm results from the balance between the mitochondrial respiratory chain (MRC) activity which pumps protons at complexes I, III, and IV from the matrix to the inner membrane space (IMS), and the F0-F1-ATP synthase which exploits the resulting proton gradients, Δp, to drive the conversion of ADP to ATP, a process called oxidative phosphorylation (OXPHOS).^12^ Under steady-state conditions, the total proton return is exactly balanced by proton pumping by the MRC, but any change in oxidative capacity that alters proton pumping could ultimately modulate the ΔΨm.^13^ Consequently, the ΔΨm is a relevant candidate sensor linking OXPHOS activity and mitochondrial dynamics by modulating OPA1 processing, in order to adapt ATP requirements and maintain cellular energy homeostasis. In fact, evidence suggests an intricate relationship between OXPHOS activity and mitochondrial structure and dynamics, but the intermediate links between these processes remain unclear. In neurological diseases due to altered mitochondrial dynamics, such as type 2A Charcot-Marie-Tooth with pathogenic *MFN2* variants and dominant optic atrophy (ADOA) with pathogenic *OPA1* variants, some defects in OXPHOS coupling and/or altered MRC activity have been found in mouse models^14^ and primary fibroblasts from patients,^15^^,^^16^ while the reverse connection linking OXPHOS activity and the network morphology, remains more obscure. In yeast and human tumoral cell lines, connected mitochondrial networks were correlated with metabolic orientation toward OXPHOS.^17^^,^^18^ However, OXPHOS dysfunctions do not systematically trigger mitochondrial fragmentation (Figure S1),^19^ as in respiratory chain disorders, mitochondria are often resistant to fission.^20^ The complete fragmentation of the mitochondrial network is a critical event, often preceding the elimination of mitochondria by mitophagy or the cell death by apoptosis. Therefore, it is essential to better understand how cells respond to mitochondrial bioenergetics alterations, and either resist to mitochondrial fission or trigger a chain of events leading to the fragmentation of the network. Yet, it remains a challenge to understand how the mitochondrial fission/fusion balance responds to bioenergetic alterations, in particular with respect to the type of mitochondrial dysfunction or stress, generated by different alterations of bioenergetic parameters and from cellular phenotypes, especially primary vs. immortalized cells.^21^ Indeed, in contrast to many tumor cell lines, healthy primary skin fibroblasts use both oxidative and glycolytic metabolisms to comply with their energetic needs,^22^ they display tubular and connected network and show similar ΔΨm between cells, and along the mitochondrial networks.^23^ Thus, fibroblast cells represent a relevant model for the exploration of mitochondrial structure-function relationships.

In this study, we used primary fibroblasts from control individuals and patients with OXPHOS defects to investigate how ATP synthesis and the mitochondrial energy balance control mitochondrial morphology. Our data highlight a crucial role of the reverse ATP hydrolase activity of F0F1-ATP synthase to maintain mitochondrial membrane potential and prevent mitochondrial fragmentation under energetic stress.

## Results

### ATP hydrolysis by F0F1-ATP synthase maintains the mitochondrial membrane potential when mitochondrial respiratory chain is inhibited

**Figure 1:**
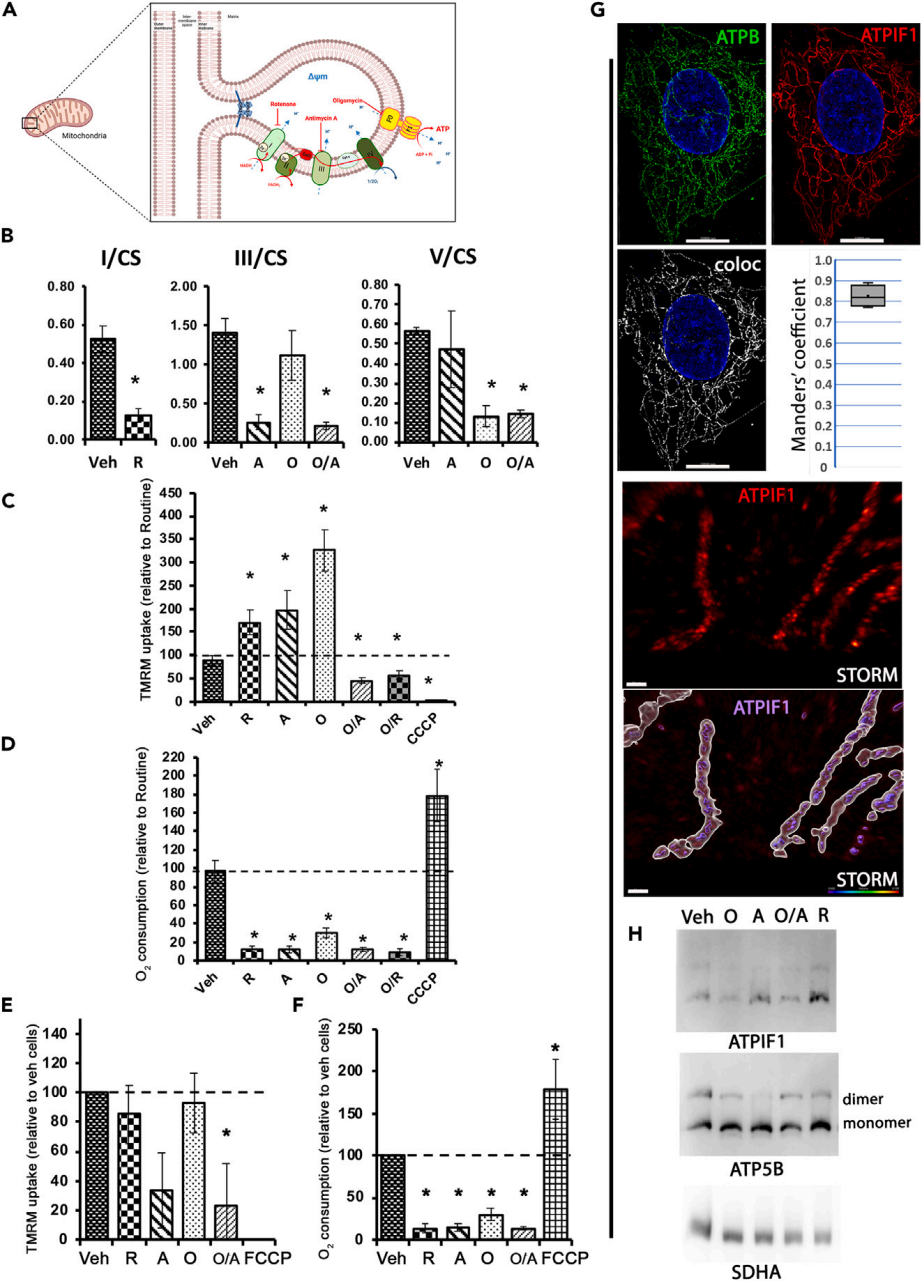
ATP hydrolysis by F0F1-ATP synthase maintains the mitochondrial membrane potential following mitochondrial respiratory chain inhibition (A) OXPHOS complexes within mitochondrial cristae. CI, CIII, and F0F1-ATP synthase are specifically inhibited by rotenone, R, antimycin, A, and oligomycin, O, respectively. (B) Enzymatic activity of OXPHOS complexes following drug application. Control fibroblasts were treated in culture conditions during 1 h, either with vehicle (Ethanol, 1/2000), R (2.5 μM), A (2 μg/mL), O (4 μg/mL), and O combined to A. CI, CIII, and ATP synthase activities were normalized to citrate synthase (CS). *n* = 4 different control cell lines, each measured in duplicates. Results are presented as means ± SEM. ∗indicates significant difference from vehicle conditions. (C and D) Combined measurement of mitochondrial membrane potential and O2 consumption in intact cells. Monitoring of TMRM fluorescence (C) coupled to oxygraphy (D) after a 30 min inhibition with either vehicle (Ethanol, 1/2000), R (2.5 μM), A (2 μg/mL), O (4 μg/mL), O combine to R and O combined to A. The CCCP (10 μM) uncoupler was used as positive controls for mitochondrial depolarization, n = 4–6 different control cells, each in duplicate. Results are expressed relative to cellular routine respiration (C) or TMRM uptake (D), i.e., delta fluorescence (see Figure S3 for detailed analysis) and presented as means ± SEM. ∗indicates significant difference (*p* < 0.05) from vehicle conditions, Mann-Whitney U test. Measurement of mitochondrial membrane potential (E) and O2 consumption on permeabilized cells (F). TMRM uptake was measured on cells treated for 30 min with the different inhibitors, *n* = 4, each in duplicate. The FCCP (10 μM) uncoupler was used as positive controls for mitochondrial depolarization. Results are presented as means ± SEM. ∗*p* < 0.05, Mann-Whitney U test. (G and H) ATPIF1 interacts with F0F1-ATP synthase and shows normal distribution within the mitochondria from human skin fibroblasts. The mitochondrial network was revealed by immunostaining of anti-ATPIF1 antibodies (Red) and anti-ATP5B (Green). The colocalization channels are shown in white produced by the IMARIS software colocalization tool with corresponding Manders colocalization coefficient. Super-resolution STORM imaging of ATPIF1 and the corresponding representation with the intramitochondrial localization of ATPIF1 in violet, using Imaris Iso-surface rendering. Blue native PAGE followed by western-blot analysis (H). Membranes were sequentially hybridized with monoclonal antibodies targeting ATPIF1 and ATP synthase subunit ATP5B, and SDHA as a control loading.

To understand the role of deficits of each respiratory chain complex in the coordination between mitochondrial bioenergetic activity and ΔΨm, we used standard inhibitors that specifically target: (1) rotenone, a CI inhibitor inhibiting NADH oxidation and antimycin A, a CIII inhibitor (Figure 1A), thereby inhibiting both NADH and FADH_2_ oxidations; (2) oligomycin, a F0F1-ATP synthase inhibitor; and (3) combined oligomycin/antimycin (O/A) and oligomycin/rotenone (O/R) treatments. When applied in the cell culture media on intact fibroblasts, these drugs efficiently and durably inhibit the targeted enzyme activities, as confirmed by activity measurements 1 h after treatment (Figure 1B).

Next, the ΔΨm and respiration rates were simultaneously measured on intact cells following rotenone, antimycin, or oligomycin treatment (Figures 1C and 1D). The drastic inhibition of the respiration rates confirmed that the inhibitors exerted the expected effects on targeted MRC complexes (˗86% and ˗85%, for rotenone and antimycin, respectively, *p* < 0.05, Figure 1D), while cells treated with the ATP synthase inhibitor retained residual respiration, not coupled to ATP synthesis (Leak respiration). Interestingly, while CI or CIII inhibition drastically inhibited electron transfer and thus proton pumping within the MRC, it did not induce a decrease in ΔΨm, as expected (Figure 1C), but a hyperpolarization (antimycin: +98% TMRM uptake compared to vehicle-treated cells; rotenone +75% TMRM, *p* < 0.05). As control, we used the mitochondrial uncoupler CCCP^28^ which did completely collapse the ΔΨm and thus increased the respiration rate (Figures 1C and 1D). The action of these drugs lasts several hours, as we showed by acquiring the TMRM signal by microscopy (Figure S2). After 4 h, the mitochondrial membrane potential was maintained under rotenone, antimycin, or oligomycin but decreased under O/A.

In physiological OXPHOS condition, the proton motive force generated by the MRC complexes I, III, and IV fuels ATP synthesis by the F0F1-ATP synthase functioning in the forward mode. Yet, it has been demonstrated that upon decrease of the ΔΨm or matrix pH, F0F1-ATP synthase can also function in its reverse mode, hydrolyzing matrix ATP into ADP and pumping H^+^ across the IMM.^29^ When F0F1-ATP synthase activity is also inhibited by oligomycin, in conjunction with that of MRC, in this case, the ΔΨm drops (Figure 1C, oligomycin + antimycin: O/A, oligomycin + rotenone: O/R (Figure S2, O/A)), while the inhibition of the F0F1-ATP synthase alone did not induce a depolarization, but, as expected, a significant hyperpolarization (Figure 1C, O, *p* < 0.05) by inhibiting the proton flow back to the matrix through the F0F1-ATP synthase. To clarify this effect, we monitored the time course evolution of mitochondrial respiration and ΔΨm: first, routine respiration and corresponding ΔΨm in this phosphorylating condition were measured, then the evolution of these two parameters, respiration rate and ΔΨm, were recorded for 30 min following blockade of MRC and/or F0F1-ATP synthase (Figure S3). Both rotenone and antimycin immediately inhibit the oxidation at MRC (Figures S3B and S3D); yet despite the MRC’s inactivity, the ΔΨm never dropped and even evolved to a slight hyperpolarization over time. Further inhibition of F0F1-ATP synthase by oligomycin led to the fall of ΔΨm within seconds when MRC was fully inhibited (CIII inhibition by antimycin, Figure S3B) and within 30 min when only CI was inhibited with rotenone (Figure S3D). Moreover, immediate inhibition of F0F1-ATP synthase, together with inhibition of MRC with either rotenone or antimycin not only prevented the hyperpolarization but, on the contrary, induced progressive depolarization (Figures S3C and S3E). Oligomycin inhibits both ATP synthase and ATP hydrolase activities of the F0F1 complex. In order to verify if the ATP-hydrolyzing mode of the F0F1-ATP synthase is involved in the observed maintenance of the ΔΨm, we forced the reaction in the forward direction, i.e., ATP synthesis. For this purpose, fibroblasts were permeabilized by digitonin to control OXPHOS substrate supply (Figures 1E and 1F). Analyses were performed in the presence of malate, pyruvate, and succinate, ensuring a fully operating TCA cycle and the supply in both NADH (CI) and FADH2 (CII) to the MRC. Then, ADP was added to stimulate ATP synthesis, thus favoring the forward mode functioning of F0F1-ATP synthase by a high ADP/ATP ratio. In this condition, MRC inhibition was similar to the one observed on intact cells as highlighted by the drastic reduction of respiration rates (Figure 1F). However, in stark contrast to the measurements performed on intact cells, under this condition where H^+^ compensation flux through ATPase reverse mode cannot occur, the ΔΨm was not maintained anymore but instead tended to decrease following antimycin treatment (−70% of TMRM uptake, *p* = 0.07) (Figure 1E).

Altogether, these results point to a role of the F0F1-ATP synthase in ATP hydrolyzing mode to maintain the mitochondrial membrane potential. In many cell types, the reverse mode is controlled by ATPIF1 action, the ATPase inhibitor. Indeed, in cardiomyocytes, neurons, and tumoral cell lines, acute MRC inhibition induces ATPIF1 activation and binding to the catalytic site of the F1 domain to block rod rotation and prevent ATP hydrolysis.^30^^,^^31^ We wanted to see whether the activation of the reverse mode in fibroblast could be linked to a difference in ATPIF1 expression or binding. Our results showed by super-resolution STORM that ATPIF1 displayed a normal distribution within the mitochondrial network and colocalizes with F0F1-ATP synthase beta subunit (Figure 1G). Blue native-PAGE experiments demonstrated colocalization of ATPIF1 with F0F1-ATP synthase, in both monomer and dimer forms of F0F1-ATP synthase (Figure 1H). Despite the expression of ATPIF1 in our primary cells, the ATP-hydrolyzing mode of the F0F1-ATP synthase occurred following MRC inhibition.

### ATP hydrolysis by F0F1-ATP synthase ensures connectivity of the mitochondrial network

**Figure 2:**
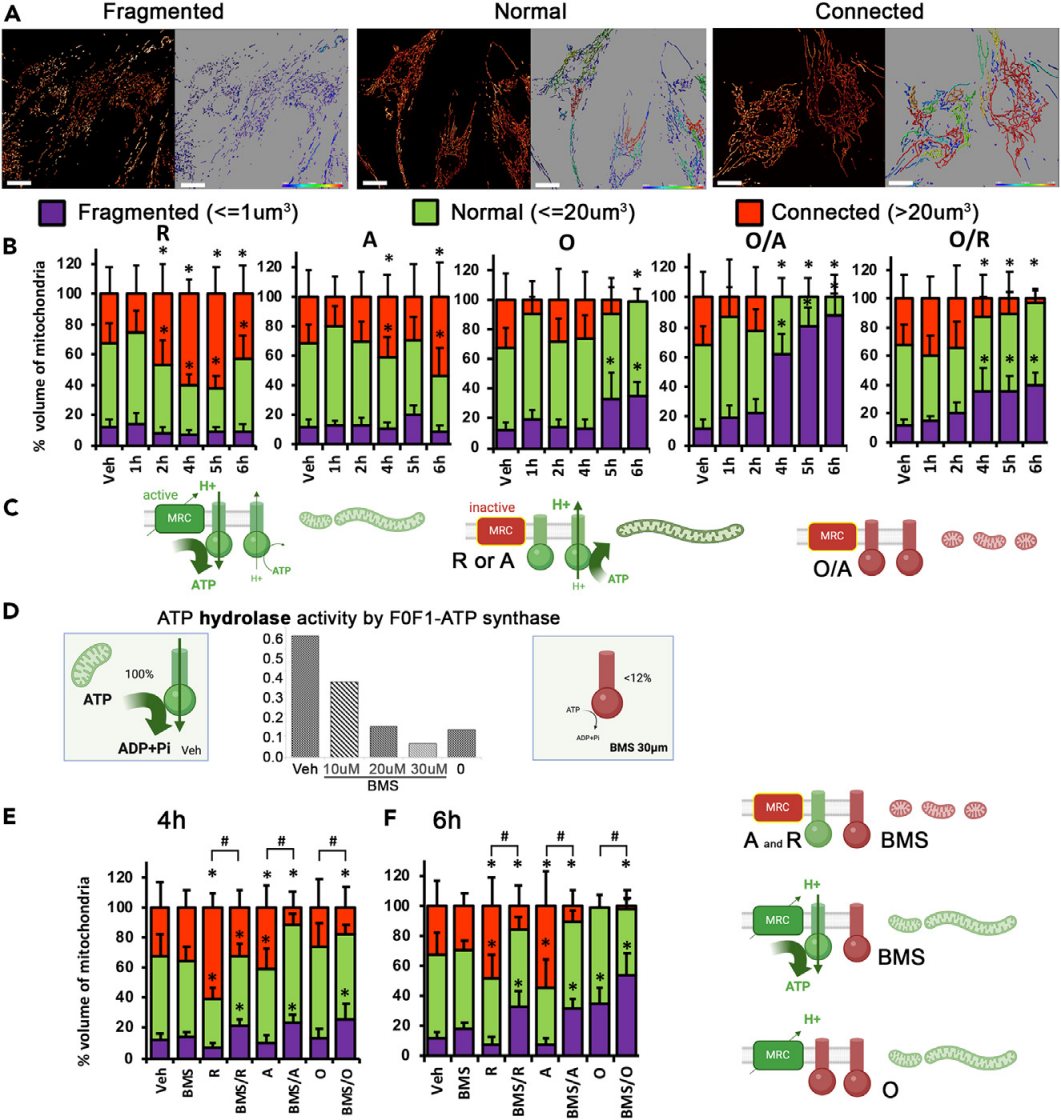
The hydrolase activity of F0F1-ATP synthase compensates for the dysfunction of respiratory chain and prevents mitochondrial fission (A) Mitochondrial network observed with MitoTracker Green by fluorescence microscopy. Left panel: representative images showing the different morphologies of the mitochondrial network. Right panel: Colorimetric representation of the mitochondrial network according to mitochondrial volume Imaris iso-surface rendering. (B) Quantification of mitochondrial volume following OXPHOS inhibition. 3D images analyses were performed using Imaris software (Bitplane). The iso-surface module quantifies the volume of mitochondria in μm^3^. Corresponding percentage of mitochondrial volume of ≤1 μm^3^ (fragmented, in violet), ≤20 μm^3^ (Normal, in green) and >20 μm^3^ (connected, in red). *n* = 3, analyses were performed on 12 images on three independent biological replicates. Results are presented as means ± SEM. ∗ indicates significant difference from control conditions (Vehicle, Veh). (C) Diagram summary under normal conditions reveal that F0F1-ATP synthase and hydrolase activities work simultaneously in mitochondria with a high proportion of forward action. Schematic representations of the changes in the activity of the complexes and shape of mitochondria following the presence of inhibitors. (D) BMS inhibition of F0F1-ATPase on human fibroblasts. Panels: schematic representation of ATP hydrolase activity following drug application. Activity of mitochondrial F0F1-ATPase (V) normalized to citrate synthase (CS). Fibroblasts were treated in culture conditions 4 h with oligomycin or different concentrations of BMS in order to define the effective BMS concentration that inhibits F0F1-ATPase activity. *n* = 2, each in duplicate. (E and F) Mitochondrial network following BMS treatments observed using MitoTracker Green by fluorescence microscopy in human control cells, treated for 4 h (E) or 6 h (F) with BMS (30 μM), BMS + R, BMS + A, or BMS + O or Veh. *n* = 5, analyses were performed on 12 images on four independent biological replicates. Results are presented as means ± SEM.∗*p* < 0.05, Mann-Whitney U test. Schematic representations of the changes in the activity of the complexes and shape of mitochondria following the presence of inhibitors with BMS or O.

Fragmentation of the mitochondrial network is generally accepted as a typical reaction of cells to MRC stress. However, the reciprocal is not systematic: many studies^19^ and our data (Figure S1) show that significant MRC damage does not lead to a pattern of individualized mitochondria; on the contrary, the networks are tubular and often connected. To go further, we investigated whether ATP hydrolysis by reverse function of ATP synthase can explain the absence of mitochondrial fission. Using fluorescent deconvolution microscopy,^28^ we found that MRC inhibition does not induce mitochondrial fission (Figures 2A–2C), but rather increased the connectivity of the mitochondrial network within 2–6 h. However, combined inhibitions of the F0F1-ATP synthase and the MRC, using either oligomycin/antimycin or oligomycin/rotenone, prevented this increased connectivity and induced a massive fragmentation of the network from 4 h onwards (Figure 2B). Oligomycin alone also causes network fragmentation, albeit later (6 h) and less pronounced than O/A or O/R combination (Figure 2B).

To confirm that inhibition of ATP hydrolysis, and not any side effect of oligomycin, is responsible for this fragmentation upon MRC inhibition, we selectively inhibited the hydrolase activity of F0F1-ATP synthase using BMS-199264 (BMS)^32^(Figures 2D–2F). While BMS strongly reduced F0F1-ATPase maximal activity (Figure 2D), BMS alone has no effect on respiration rate, mitochondrial membrane potential (Figure S4), or on the mitochondrial network, neither at 4 h nor at 6 h (Figures 2E and 2F). A combined inhibition of MRC and F0F1-ATP hydrolase activity with BMS, on the other hand, induces mitochondrial fragmentation (Figures 2E and 2F, BMS/R, BMS/A, compared to R or A alone or to vehicle), thus implying that the reverse mode of F0F1-ATPase is required to preserve the mitochondrial network connectivity. Interestingly, BMS in conjunction with oligomycin also increased network fragmentation, suggesting that even in the absence of respiratory chain inhibition, F0F1-ATPase activity takes part in mitochondrial dynamics.

**Figure 3:**
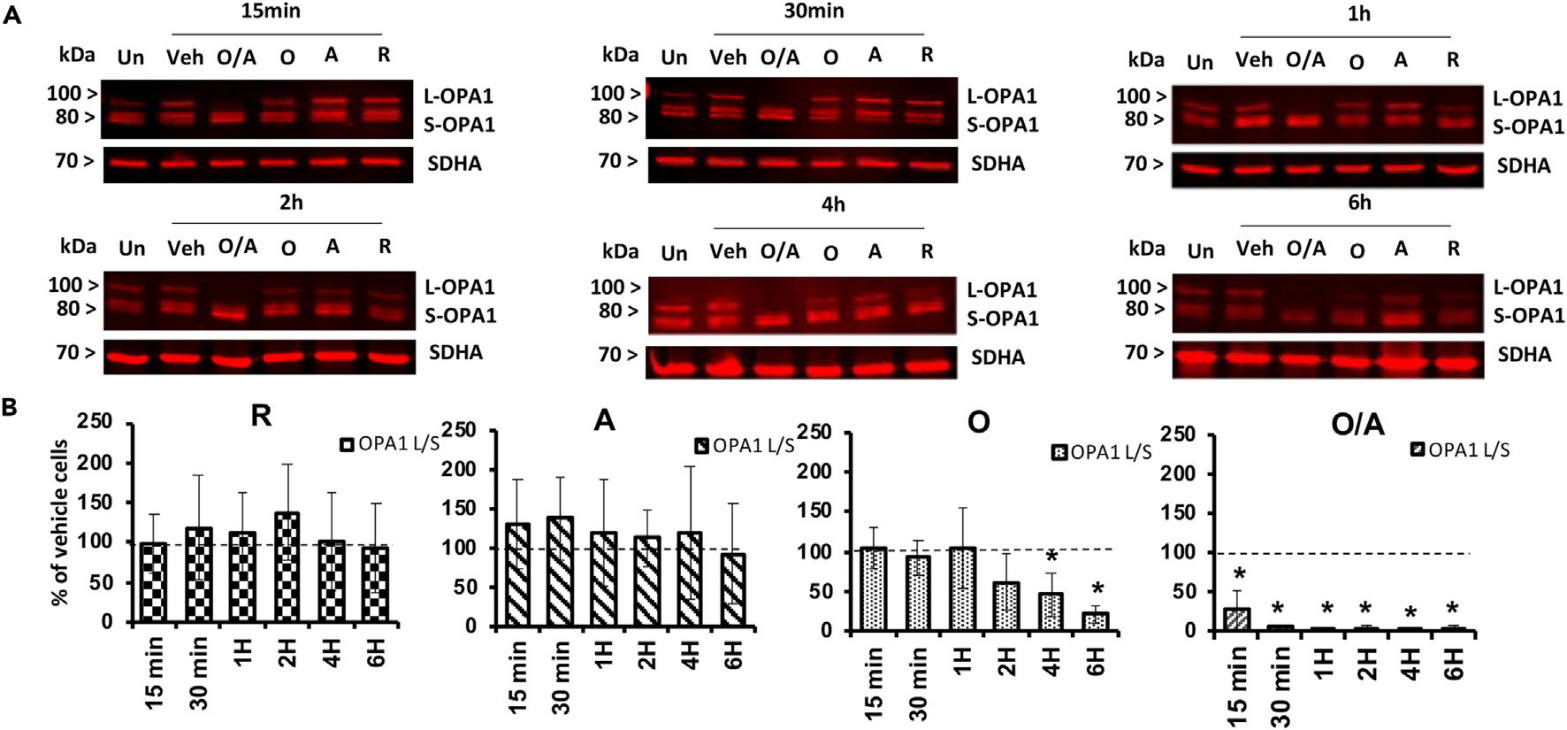
Effect of OXPHOS inhibition on OPA1 protein cleavage (A) OPA1 isoforms’ expression profile: Representative images of Western-blots of OPA1 long (L) and short (S) isoforms and SDHA (mitochondrial loading reference) performed on protein extracts from treated cells. (B) Quantification of the OPA1 L/S ratio relatively to the vehicle. *n* = 5, in five independent biological replicates. Results are presented as means ± SEM. ∗ Indicates significant difference from control conditions (Veh).

A key player of mitochondrial dynamics is the inner membrane dynamin OPA1^33^ and more specifically the equilibrium between the L-OPA1 and S-OPA1 isoforms In agreement with the analysis of the mitochondrial network structure, we did not observe any cleavage of OPA1 following antimycin or rotenone exposure (Figures 3A and 3B). Strikingly, combined inhibitions of the MRC and the F0F1-ATP synthase using O/A induced rapid L-OPA1 cleavage as early as 15 min, with S-OPA1 being the unique isoform detectable after 1 h of treatment (Figures 3A and 3B). This rapid S-OPA1 accumulation was followed, but largely preceded, the mitochondrial fission, leading to a massive fragmentation of the network at 6 h (Figure 2B). In OMA1^−/−^ MEFs, joint inhibition of MRC and the F0F1-ATPase did not induce OPA1 protein cleavage while OPA1-L cleavage occurred in WT MEFs (Figures 5A and 5B). Consistently, although the mitochondrial network in OMA1^−/−^ MEFs cells is more fragmented than in WT MEFs *per se,* we do not observe any further fragmentation following O/A treatment (Figure S5C) while WT MEFs treatment was accompanied by a rapid and massive fragmentation of the mitochondrial network. These results point to the role of the ΔΨm-sensitive OMA1 protease in OPA1 processing leading to mitochondrial fragmentation.

Overall, our data indicate a key role of F0F1-ATPase reverse mode in the resistance to fission of the mitochondrial network upon MRC inhibition, through maintenance of ΔΨm, thereby preventing L-OPA1 cleavage. However, these data also suggest that F1F0 complex itself may regulate mitochondrial network dynamics, as L-OPA1 protein cleavage was also observed after oligomycin treatment alone, at 4 h (Figure 3), preceding partial mitochondrial fragmentation (Figure 2B).

### Inhibition of mitochondrial network fission relies on glycolytic energetic metabolism

**Figure 4:**
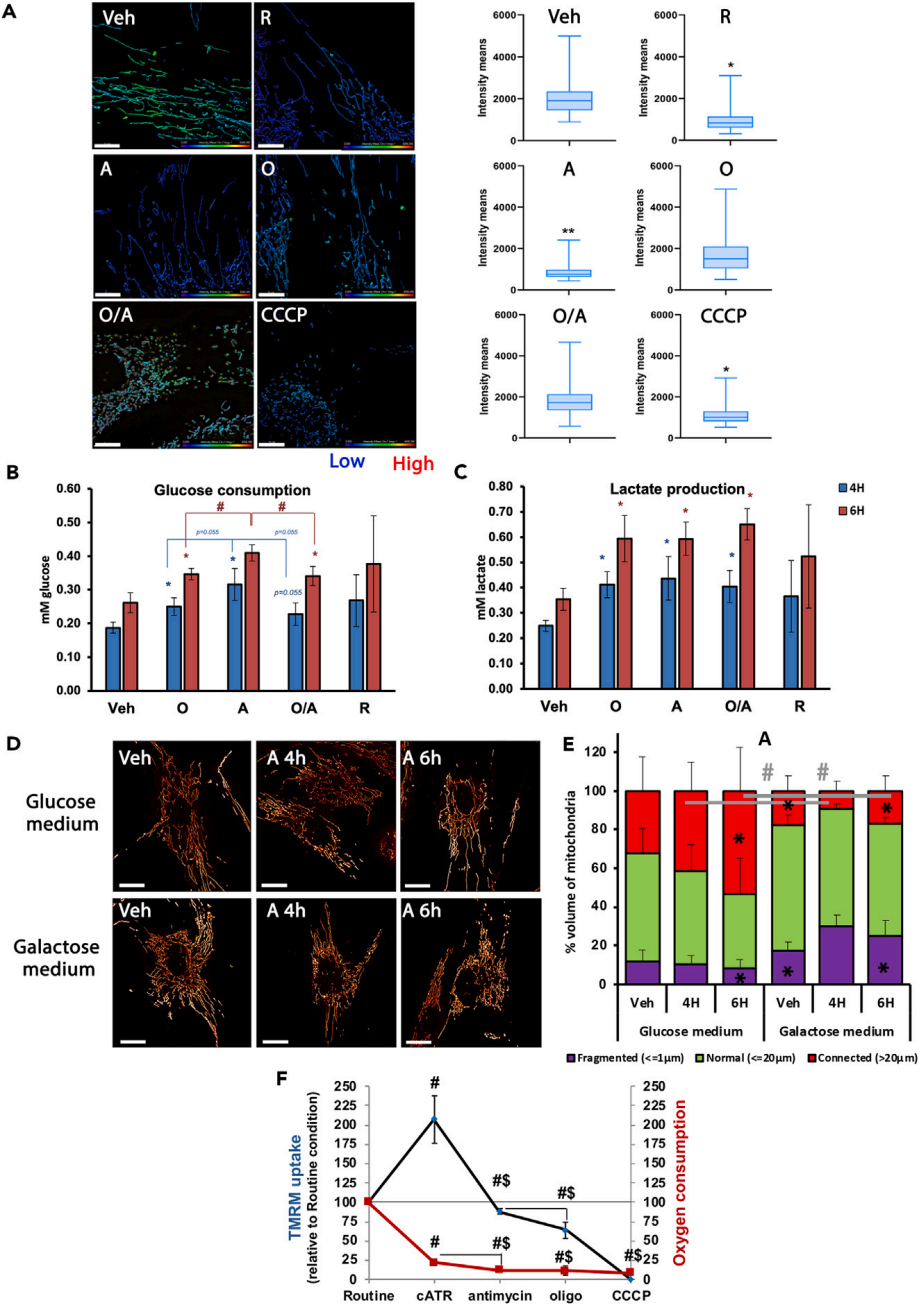
Glycolytic metabolism supports the resistance to fission in response to respiratory chain dysfunction (A) Measurement of mitochondrial ATP using the ATP Red probe by fluorescence microscopy in control fibroblasts after 4 h inhibition of MRC (antimycin, A or rotenone, R), F0F1-ATP synthase inhibition (oligomycin, O) or both (oligomycin/antimycin, O/A) or uncoupler CCCP. *n* = 3, in duplicates. (B and C) Measurement of the cellular glycolytic metabolism. Cellular glucose consumption and lactate production were measured in vehicle-treated cells, or after MRC inhibition (antimycin, A or rotenone, R), F0F1-ATP synthase inhibition (oligomycin, O) or both (oligomycin/antimycin, O/A). Results are presented in mM glucose per 4 h and mM Lactate per 4 h in blue, in mM glucose per 6 h and mM lactate per 6 h in red. *n* = 4, in duplicates. Results are presented as means ± SEM.∗ Indicates significant difference from vehicle conditions, # indicates significant difference among inhibitors, using the Mann-Whitney U test. (D) Mitochondrial network observed using MitoTracker Green by fluorescence microscopy in control fibroblasts treated 4 h and 6 h with Antimycin (2 μg/ml) in DMEM F12 medium or in galactose medium. (E) Quantification of mitochondrial volume following CIII inhibition in galactose medium. The percentage of mitochondria length represents the percentage of ≤1 μm (fragmented, violet), ≤20 μm (normal, green) and >20 μm (connected, red) mitochondria, *n* = 3, in three replicate. Results are presented as means ± SEM.∗ indicates significant difference from vehicle conditions, # indicates significant difference among media, using the Mann-Whitney U test. (F) Modulation of the mitochondrial membrane potential and respiration rates over time in intact cells after adenine nucleotide transporter (ANT) inhibition. TMRM uptake (black trace) as well as the corresponding routine respiration rate (O2 flux, red trace) were first recorded for 2–3 min in routine condition using Oroboros technology. ANT was then inhibited using 30 μM carboxyatractyloside (cATR), followed by antimycin injection. TMRM uptake and respiration rate were recorded again after each inhibitor addition. CCCP was used to fully depolarize mitochondria. Results are presented as means ± SEM (*n* = 3). # indicates significant difference from routine conditions, $ indicates significant difference between inhibitors, using the Wilcoxon test for paired data.

F1F0-ATP hydrolase activity would imply an increased intra-mitochondrial ATP consumption. To determine mitochondrial ATP content after inhibition of MRC and/or F0F1-ATP synthase, BioTracker ATP-Red fluorescence microscopic imaging was used^26^ (Figure 4A). As expected, in the presence of the uncoupler CCCP, the ATP-Red signal was significantly lower than in control conditions, while inhibition of F0F1-ATP synthase alone by oligomycin did not significantly reduce the ATP-Red fluorescence (Figure 4A, −13%, *p* = 0.32). Interestingly, the intra-mitochondrial ATP levels were lowest in cells that were treated with antimycin or rotenone (−55 and −52%, respectively, *p* < 0.05). These fluorescence changes, when MRC is inhibited, were indeed due to F0F1-ATPase activity, since they were abolished by the ATP synthase inhibitor oligomycin (Figure 4A). This result strongly supports intra-mitochondrial ATP hydrolysis via F0F1-ATPase activity when MRC is inhibited. To test whether this ATP consumption is supported by glycolytic ATP production, we assessed the glycolytic metabolism by measuring cellular glucose consumption and lactate production (Figures 4B and 4C). As expected, combined MRC and the F0F1-ATP synthase inhibitions impair mitochondrial ATP synthesis and stimulate glycolytic metabolism, as revealed by increased glucose consumption and lactate production. However, glucose consumption was higher in antimycin-treated cells than oligomycin or O/A-treated ones. All of these drugs inhibit oxidative metabolism and oxidative phosphorylation, which implies an almost complete dependence on glycolytic metabolism for ATP production, regardless of which inhibitor is used. Therefore, the increased glucose consumption in antimycin-treated cells strongly suggests greater cellular ATP requirement consistent with mitochondrial ATP hydrolysis of the F0F1-ATP synthase.

In galactose medium, glycolysis is strongly slowed down^34^ and becomes limiting for cellular ATP production, which consequently relies mainly on oxidative phosphorylation.^35^ In this respect, galactose medium sensitized mitochondria to the fragmentation caused by inhibition of MRC, as shown by the significant increase of the mitochondrial fragmentation after 4 h or 6 h of antimycin treatment, compared to both glucose medium treatments or vehicle condition (*p* < 0.05, Figures 4D and 4E). The production of glycolytic ATP to support the mitochondrial ATP hydrolysis requires the import of cytosolic ATP, via adenine nucleotide translocase (ANT), into the mitochondrial matrix. We studied the role of this ATP/ADP carrier using oxygraphy coupled to the measurement of ΔΨm by sequential inhibition of ANT using cATR, followed by inhibition of MRC by antimycin and finally inhibition of F0F1-ATP (Figure 4F). ANT inhibition progressively inhibited the phosphorylating respiration, as expected, through inhibition of ADP import (Figure 4F) and simultaneously hyperpolarized mitochondria. This inhibition of ATP/ADP exchange prevents the maintenance of ΔΨm upon MRC proton pumping arrest (Figure 4F). F0F1-ATPase inhibition further decreased the ΔΨm. Altogether these data confirm that resistance to fission relies, at least in part, on glycolytic metabolism providing ATP within mitochondria for F0F1-ATPase activity. In addition, the ATP^4−^ imported into mitochondria across the ANT in exchange for ADP^3−^ may also contribute to the maintenance of the ΔΨm through the gain of a negative charge on the matrix side.

### F0F1-ATP synthase mutants are prone to MRC-induced mitochondrial fission

**Figure 5:**
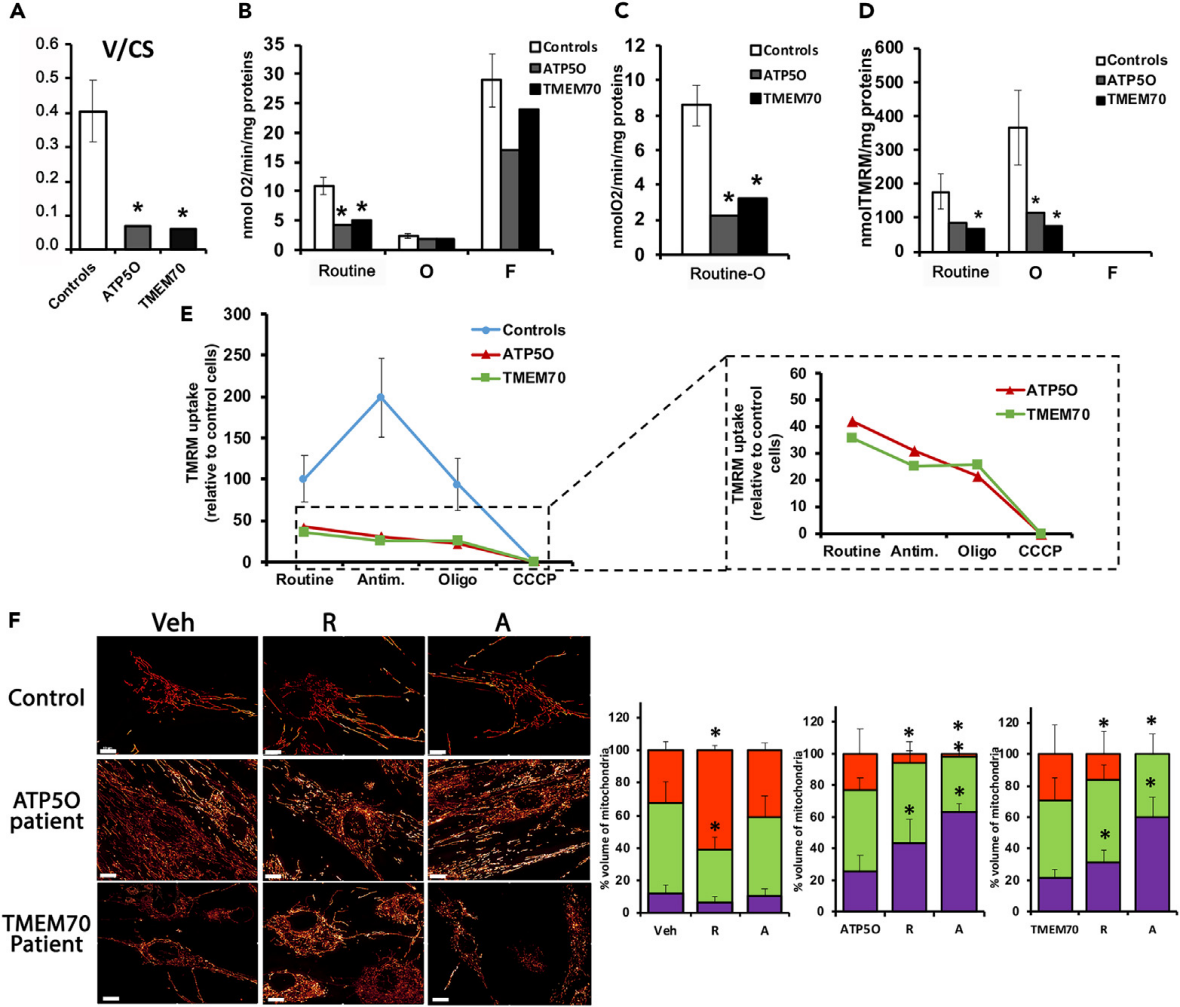
Genetic deficiency of the F0F1-ATP synthase sensitizes cells to mitochondrial fission (A) Activity of F1-ATPase (V), normalized to citrate synthase (CS), measured in controls and in mutant cell lines. (B–D) Combined measurement of mitochondrial membrane potential and O2 consumption on intact control and patient cells. (B) Measurement of respiration rates using Oroboros technology. Routine respiration measured on intact cells in culture medium and corresponding to oxidative metabolism. Non-phosphorylating respiration rate (O), measured after inhibition of ATP synthesis through addition of oligomycin (4 μg/mL). Maximal oxidation capacity (F), determined after progressive uncoupling through FCCP titration (for detailed analysis and injection sequence, see Figure S6). (C) Phosphorylating respiration rate, Routine-O: routine minus oligomycin respirations, i.e., respiration dedicated to ATP synthesis. (D) Mitochondrial membrane potential in intact patient cells. TMRM uptake was measured on control and patient intact cells in routine condition and in non-phosphorylating condition after F0F1-ATP synthase inhibition by oligomycin (O) in the same sample as in (B). Oxygen consumption rates and TMRM uptake were normalized to cellular protein concentration. Control cells: *n* = 6, Results are presented as means ± S.D. ATP5O and TMEM70-mutated cells: 3 independents replicates (inter-assay C.V. for ATP5O: Routine, 7.4 and 10.6%; Oligo, 6.5 and 20.8%, FCCP: 9.9 and 0% for respiration rates and TMRM measurements, respectively. TMEM70: Routine, 14.4 and 29.1%; Oligo, 27.1 and 17.8%, FCCP: 21.0 and 0% for respiration rates and TMRM measurements, respectively). ∗ Indicates significant difference from control conditions (value beyond 2 S.D. from controls). (E) Modulation of the mitochondrial membrane potential in controls and F0F1-deficient cells. TMRM fluorescence was first measured in routine condition, then 30 min after injecting antimycin (A: 2 μg/ml), and then after adding oligomycin (O: 4 μg/ml; ATP synthase inhibitor). CCCP was used as a control to fully depolarize mitochondria. Control cells: *n* = 5, ATP5O and TMEM70-mutated cells: 3 independents replicates. Results are presented as mean ± SD. The selection of patient curves allows a change of scale (Right panel). (F) Mitochondrial network observed with MitoTracker Green by fluorescence microscopy. Left panel: representative images of the different morphologies of the mitochondrial network observed following MRC inhibition in patient fibroblast cells. Controls and ATP5O and TMEM70 patient fibroblasts were treated 4 h with rotenone (R: 2.5 μM) or with Antimycin (A: 2 μg/mL). Right panel: corresponding quantification, the percentage of mitochondria volume represents the percentage of ≤1 μm (fragmented, violet), ≤20 μm (normal, green) and >20 μm (connected, red) mitochondria. Controls, *n* = 3, patient cells: analyses were performed on 12 images on triplicates. Results are presented as mean from each patient relative to control cells. ∗ Indicates significant difference from Ctrl (value beyond 2 S.D. from controls).

We then investigated the sensitivity of F0F1-ATP synthase mutants to mitochondrial fission using genetic models. Fibroblasts from patients with mutations on the ATP synthase peripheral stalk subunit ATP5O and on the F0F1-ATP synthase assembly factor TMEM70 (Table S1) display a drastic complex V deficiency, with a residual F0F1-ATPase maximal activity of 18% and 16%, respectively (Figure 5A). F0F1-ATP synthase assembly and supramolecular assembly into dimer were also severely impaired (Figures S6B–S6D). Oxygen consumption measurements confirmed the significant decrease in the routine respiration (Figures 5B, 5C, and S6A) and a strong decrease of the phosphorylating respiration (Routine-0) in ATP5O and TMEM70 mutants (−60% both). It is noteworthy that in routine conditions the mitochondrial membrane potential was significantly reduced (∼−60%, beyond 2 SD to the control mean, Figure 5D). In addition, F0F1-ATP synthase inhibition by oligomycin, which induced hyperpolarization in control cells, had no effect on mutant cells (Figure 5D). It appeared interesting to investigate whether these genetic models of F0F1-ATP synthase deficiency could nonetheless correct the ΔΨm following inhibition of MRC.

We monitored over time how ΔΨm varies in response to MRC inhibition. Our results showed that both mutants cannot sustain their ΔΨm following MRC inhibition: antimycin led to a decrease of the ΔΨm whereas the addition of oligomycin had no further effect (Figure 5E). Interestingly, mitochondrial network analysis by fluorescence microscopy did not show any significant difference between the mutant and the control cell lines (Figure 5F). However, both F0F1-ATP synthase mutants cannot sustain MRC proton pumping arrest, as witnessed by a fragmentation of the mitochondrial network. The effect was even stronger with a complete OXPHOS inhibition using antimycin (ATP5O: 62.71%; TMEM70: 59.79% fragmented mitochondria with antimycin vs. control: 10.20% with antimycin, *p* < 0.05) (Figure 5F). Mutants also showed a constitutive higher rate of OPA1 processing, particularly in TMEM70 mutant (Figure S7). This may be related to the lower mitochondrial membrane potential and could sensitives these cells to MRC inhibition. Taken together, these results confirm that MRC-induced ΔΨm decrease drives mitochondrial fission only when the compensation by F1-mediated-ATP hydrolysis is inhibited.

## Discussion

### Defects in the respiratory chain do not lead to fragmentation of mitochondria

One striking property of the mitochondrial network is that it can rapidly shift from a fused to a fragmented state when cells experience metabolic or environmental stresses, which further enables the selective removal of damaged organelles by mitophagy.^36^ Therefore, given the links between mitochondrial dynamics and quality control, it is likely that the balance between fission and fusion and subsequently, mitophagy, is coordinated to cellular OXPHOS activity. However, despite major alterations of OXPHOS activities and respiration rates,^35^^,^^37^^,^^38^ cells displaying MRC deficiencies often demonstrate unaltered mitochondrial network^19^^,^^20^ (Figures S1A and S1B) and no significant drop in their ΔΨm (Figure S1E). This raises questions about the underlying mechanisms linking OXPHOS activity and mitochondrial fission, and how mitochondria in these conditions can resist to fission. Cells from patients with genetic MRC defects represent models of chronic mitochondrial stress but do not allow the study of responses occurring prior to metabolic adaptation or elimination of damaged mitochondria by mitophagy. Here, we used chemical drugs whose immediate inhibition of the MRC or the ATP synthase enabled us to control the bioenergetic stress initiation, while we monitored the subsequent early change in the mitochondrial morphology. Our findings indicate that the mitochondrial network connectivity resists to MRC inhibition. These results are also in agreement with previous studies which do not show fragmentation of the mitochondrial network following MRC inhibition,^19^^,^^20^ while conversely, we showed that MRC inhibition leads to hyperpolarized and hyperfused mitochondria. Cellular stress, such as serum or amino acid deprivation or acidosis has previously been shown to increase mitochondrial length.^39^^,^^40^ This process was named stress-induced mitochondrial hyperfusion (SIMH) and represents an adaptive pro-survival response against stresses requiring L-OPA1 isoform. Decreased ΔΨm promotes L-OPA1 isoforms cleavage to S-OPA1 by activation of OMA1 proteases^41^^,^^42^ and further inhibits the fusion of the inner membrane and induces fission of the mitochondria. However, whether ΔΨm hyperpolarization can promote L-OPA1 stability and increase mitochondrial fusion remained to be determined.

### F0F1-ATP synthase is the primary sensor of mitochondrial damage and inhibition of MRC immediately activates mitochondrial ATP hydrolase activity to prevent fission

ΔΨm level results from OXPHOS and proton pumping activity of complexes I, III, and IV. Under physiological condition where the electrochemical proton potential is sufficient to surpass the free energy of ATP hydrolysis, Δp is used to catalyze ATP synthesis which is the central function of F0F1-ATP synthase. Under steady-state conditions, backward H^+^ flux through ATP synthase and forward H^+^ through MRC are balanced. However, when the electrochemical potential decreases, as in cases of respiratory chain dysfunctions, this complex can also function as a proton pump using ATP hydrolysis. The hydrolysis of ATP by the F1 part forces the central rod to rotate counterclockwise, transferring the motion to the F0 part. This rotation results in the proton pumping from the matrix to the cristae intermembrane space,^32^ maintaining a stable ΔΨm, when it is not sustained by MRC activity.^43^ Recent evidence also suggests that the forward and reverse F0F1 activities co-exist within single mitochondria.^44^ While the majority of cristae synthesize ATP, some cristae hydrolyze ATP, a process that explains the heterogeneity of the mitochondrial membrane potential in between cristae.^44^^,^^45^ Here, we demonstrated in human fibroblasts that complete inhibition of MRC promotes mitochondrial import of cytosolic ATP and ATP hydrolysis in the matrix by the F0F1-ATPase reverse activity, preventing ΔΨm drop and mitochondrial fission. This protective effect may be due to reverse pumping of the H^+^ through F0 ATPase and, at least in part, be supported by the electrogenic ATP^4−^/ADP^3−^ exchange by ANT. However, the use of a specific inhibitor of this F0F1-ATPase activity, BMS-199264,^32^ overcomes this resistance in MRC-inhibited cells, further supporting the role of the F0F1-ATPase activity in preventing mitochondrial fission. This result is further strengthened by genetic models of deficient F0F1-ATP synthase. Our results are in agreement with those obtained previously in intact HEK293,^46^ disclosing that the reverse mode of F0F1-enzymatic activity efficiently maintains the ΔΨm.

ATPIF1 is an inhibiting factor preventing the consumption of mitochondrial ATP upon MRC injury, by limiting F0F1-ATPase activity,^30^^,^^31^ although it is not clear how ATPIF1 is activated and effective on the F0F1-ATP synthase. In primary fibroblasts, ATPIF1 is expressed and bound to F0F1-ATP synthase, but its expression was insufficient to inhibit ATPase activity. Interestingly, ATPIF1 overexpression in some cancer cell lines prevents ATP hydrolysis associated with impaired mitochondrial function and contributes to ATP homeostasis.^47^^,^^48^ As cancer cells undergo mitochondrial fragmentation following MRC inhibition,^49^^,^^50^^,^^51^^,^^52^^,^^53^^,^^54^ it suggests that if ATPIF1 prevents ATP hydrolysis, ΔΨm cannot be maintained by reverse H^+^ pumping and cells become prone to mitochondrial fission. This could partly explain discrepancies of previously published studies, which exhibited unique mitochondrial properties and morphologies according to cell types and models. However, mitochondrial dynamic regulation may depend on culture conditions,^19^ as sustained MRC inhibition and reverse F0F1-ATPase activity must be compensated by the mitochondrial import of ATP produced by glycolytic metabolism.^46^^,^^55^ The mitochondrial matrix pH below 6.5 is a critical factor for the inhibitory binding of ATPIF1 to the F1-subunit.^56^^,^^57^ Thus, as long as the glycolytic activity is supporting ATP hydrolysis and proton pumping by the F0F1-ATPase, the matrix pH would not decrease to a threshold activating ATPIF1. It is only when both glycolysis and respiration are inhibited, such as during chemical ischemia, that ATPIF1 would show its ability to attenuate cellular damage.^58^ When cultivated in 2 g/L glucose, such as in our study, instead of high 4.5 g/L concentrations, fibroblasts display an oxido-glycolytic metabolism and are consequently able to switch from OXPHOS to glycolysis, according to MRC function.^22^ Here, we found a significant increase in cellular glucose consumption following complex III inhibition, even stronger than after F0F1-ATP synthase inhibition. Moreover, in conditions forcing cellular oxidative metabolism and limiting glycolytic capacity, cells become prone to fission following MRC inhibition. Thus, these results suggest that the metabolic capabilities of different cells or tissues limit mitochondrial resistance to fission. Tissues with high oxidative metabolism such as the heart^59^ or neurons^60^ could therefore be more sensitive to MRC inhibition and fission than those favoring glycolysis^49^^,^^61^ or encompassing higher injury due to ATP hydrolysis.^62^ These metabolic changes would also affect the efficiency of the pro-fission DRP1 dynamin, as regulatory pathways as the AMPK pathway, which is sensitive to ATP/ADP/AMP ratios, promotes the recruitment of phosphorylated DRP1.^63^

Functional individualization of cristae^45^ with their own ΔΨm level opens the possibility of local depolarization responsible for mitochondrial fission. But, if local ΔΨm decrease is compensated by a reverse activity of the F0F1-ATPase, the fission signal vanishes. Our results show that despite the relevant presence of ATPIF1, some specific metabolic conditions do not prevent the F0F1-ATPase reverse activity. As cells continuously promote hyperconnectivity by systematically controlling the ΔΨm, they can mask a mitochondrial defect and prevent the elimination of defective mitochondria by mitophagy. In mitochondrial diseases, this represents a therapeutic challenge. Recent works described that ATPIF1 expression decreases in fibroblasts from patients with MRC and ATP synthase deficiencies, and revealed that epicathechin, an ATP hydrolase inhibitor, restores mitochondrial ATP content.^44^ Our results further indicate that a selective inhibitor of F0F1-ATP hydrolase also promotes deficient mitochondrial fission. Whether this fragmentation actually promotes the elimination of damaged mitochondria remains to be demonstrated, however, F0F1-ATPase appears as a promising new therapeutic target in the treatment of respiratory chain disorders.

We also found that blocking F0F1-ATP synthase by oligomycin leads to L-OPA1 processing and fission independently of ΔΨm decrease. Several hypotheses are possible, in connection with the marked increase in the ΔΨm induced by F0F1-ATP synthase inhibition, since it has been shown that hyperpolarization can also activate OMA1 and trigger L-OPA1 processing.^64^ Another link could take place following the results of Mick et al.^65^ that further demonstrated in myotubes that ATP synthase inhibition triggers Integrated stress Response (ISR) through hyperpolarization. However, the hyperpolarization induced by inhibition of the respiratory chain is less and does not lead to fragmentation, but rather to elongation of the network. This dichotomy between ISR activation/fragmentation and, on the contrary, stress-induced mitochondrial hyperfusion could be due to the intensity of hyperpolarization.

Moreover, we cannot exclude that F0F1-ATP synthase, either by its activity or by its superstructure, may play a direct role in the fission of the inner mitochondrial membrane. The involvement of F0F1-ATP synthase in cristae structuring is well-known,^66^ and the blockage of this function disrupts the remodeling of internal membranes and promotes mitochondrial fission. Preliminary results suggest that oligomycin has a direct impact on the oligomerization state of F1F0-ATP synthase, which would explain the induction of fission. Salewskij et al.^67^ demonstrated that a change in activity of the F0F1-ATP synthase correlates with its dimeric/monomeric ratio and their results support the existence of different subpopulations of complex V, ATP synthase, and ATP hydrolase. The regulation of this ratio remains to be clarified by further studies.

In conclusion (Figure S8), our results promote the F0F1-ATP synthase as a crucial actor governing mitochondrial fission triggering, at least in primary cellular models. How this mechanism takes place *in vivo* in different tissues and according to their metabolic requirement remains to be investigated.

### Limitations of the study

Our results demonstrate that the F0F1-ATP synthase is sensing the inhibition of MRC activity by immediately promoting its reverse mode of action to hydrolyze matrix ATP and restoring the mitochondrial membrane potential, thus preventing fission. Strikingly, this ATP-hydrolyzing mode was not inhibited by the ATPase inhibitor ATPIF1. However, this study was carried out on primary cells. One of the limitations of this study is whether the proposed model can be generalized to different cell types, in particular tumor cells, which often overexpress the F1-ATPase inhibitory factor, IF1. We also found that F0F1-ATP synthase inhibition by oligomycin leads to L-OPA1 processing and fission independently of ΔΨm decrease. Thus, we cannot exclude that F0F1-ATP synthase, either by its activity or by its superstructure, may play a direct role in the fission of the inner mitochondrial membrane. The link between hyperpolarization, oligomerization state of F1F0-ATP synthase, remodeling of internal membranes, and mitochondrial fission remains to be clarified by further studies.

## STAR★Methods

### Key resources table

**Table:** 

REAGENT or RESOURCE	SOURCE	IDENTIFIER
**Antibodies**		

OPA1	BD Bioscience	Cat# 612606 RRID:AB_399888
Anti-DRP1 [3B5]	Abcam	Cat# ab56788
Anti-SDHA [2E3GC12FB2AE2]	Abcam	Cat# ab14715 RRID:AB_301433
Anti-ATPase Inhibitory Factor 1/IF1 [5E2D7]	Abcam	Cat# ab110277
Anti-ATP5A [7H10BD4F9]	Abcam	Cat# ab110273 RRID:AB_10858175
Anti-ATPB [3D5]	Abcam	Cat# ab14730 RRID:AB_301438
Goat Anti-Mouse IgG H&L (Alexa Fluor® 680) preadsorbed	Abcam	Cat# ab186694
Goat Anti-Rabbit IgG H&L (Alexa Fluor® 790) preadsorbed	Abcam	Cat# ab186697
Horseradish peroxidase-conjugated α-mouse IgG	GE Healthcare UK limited	Cat# LNA931V/AH
Horseradish peroxidase-conjugated α-rabbit IgG	GE Healthcare UK limited	Cat# LNA932V/AH

**Chemicals, peptides, and recombinant proteins**		

DMEM-F12	Pan biotech	#P04-41250
DMEM, no glucose, no L-Glutamine, no sodium pyruvate	Pan biotech	#P04-01548S1
DMEM, w: 4.5 g/L Glucose, w/o: L-Glutamine, w/o: Sodium pyruvate, w: 3.7 g/L NaHCO3	Pan biotech	#P04-03500
Amniomax	Gibco	#10744274
Fetal bovin serum (FBS)	Pan biotech	#P40-37500
GlutaMAX™ Supplement	Gibco	35050038
MEM Non-Essential Amino Acids Solution (100X)	Gibco	11140035
Sodium pyruvate	Gibco/sigma	#11360-093/P2256
Uridine	Sigma Aldrich	U3750
Digitonin	Sigma Aldrich	D141
Malate	Sigma Aldrich	M1000
Succinate	Sigma Aldrich	S3674
Galactose	Sigma Aldrich	G5388
ADP	Sigma Aldrich	01905
Rotenone	Sigma Aldrich	R8875
Antimycin A	Sigma Aldrich	A8674
Oligomycin	Sigma Aldrich	O4876
FCCP	Sigma Aldrich	C2920
CCCP	Sigma Aldrich	C2759
BMS-199264 Hydroxychloride	Sigma Aldrich	BM0017
TMRM	Sigma/Molecular Probes	T5428/Cat# T668
Carboxyactractyloside (cATR)	Sigma Aldrich	PHL84196
ATP-Red Live Cell Dye	MerckMillipore	Cat#SCT045

**Critical commercial assays**		

Kit BCA Protein Assay	FisherScientific	Cat# 10678484
Glucose Colorimetric Detection Kit	Thermo Fisher Scientific, InvitrogenTM	Cat #EIAGLUC

**Experimental models: Cell lines**		

Primary human Fibroblasts	Skin biopsies (Research Ethics Committee of Angers University Hospital)	
Primary human Fibroblasts ATP5O		
Primary human Fibroblasts TMEM70		
Mouse embryonic Fibroblasts wild-type	Max-Planck-Gesellschaft zur Förderung der Wissenschaften e.V., Thomas Langer, of the Max Planck Institute for Biology of Aging	
Mouse embryonic Fibroblasts OMA1 knockout Oma1−/− KO Mouse		Anand et al., 2014^7^
Mouse embryonic Fibroblasts YME1L knockout		PMCID: PMC3998800
Mouse embryonic Fibroblasts YME1L/OMA1 double knockout		https://doi.org/10.1083/jcb.201308006

**Software and algorithms**		

Microsoft Excel	Microsoft	https://www.microsoft.com/fr-fr/
Metamorph	Molecular Device	https://fr.moleculardevices.com
Huygens Essential	Scientific Volume Imaging, Hilversum	https://svi.nl
Imaris 8.0	Bitplane	https://imaris.oxinst.com
Image Studio	LI-COR Biosciences	https://www.licor.com/bio/
OROBOROS DatLab	Oroboros Instruments	https://www.mitoeagle.org/index.php/Oroboros_Instruments
Biorender	Biorender	https://www.biorender.com

### Resource availability

#### Lead contact

Further information and requests for resources and reagents should be addressed to the main contact, who will respond, Arnaud Chevrollier (arnaud.chevrollier@univ-angers.fr).

#### Materials availability

This study did not generate new unique reagents.

#### Data and code availability

• All data reported in this paper will be shared by the lead contact upon request.
• This paper does not report original code.

### Experimental models and study participant details

#### Primary human fibroblasts

Primary fibroblasts were obtained from skin biopsy from 10 individuals, 2 patients with mitochondrial MRC deficiency and 2 patients with F0F1-ATP synthase deficiency and 7 healthy controls (Table S1). Written informed consent was obtained from all participants (Ethics Committee from the Angers University Hospital approval: CPP Ouest II – Angers, France; Identification number: CPP CB 2014/02; Declaration number: DC-2011-1467 and Authorization number: AC-2012-1507). Primary human fibroblasts were cultured in 2/3 Dulbecco’s modified Eagle medium (DMEM-F12, PAN Biotech, Wimborne, UK), 1/3 Amniomax (Gibco, Invitrogen, Paisley, UK) (glucose concentration, 2 g/L and glutamine, 1 mM) supplemented with 10% fetal bovine serum (PAN Biotech, Wimborne, UK), 1 mM sodium pyruvate (Gibco, Invitrogen, Paisley, UK), and 50 μg/mL uridine (Sigma Aldrich, Lyon, France) at 37°C, 5% CO2. All experiments were conducted on fibroblast cultures between passages 6 and 25 to avoid artifacts due to senescence.^24^

#### Cell line

Mouse embryonic fibroblasts MEFs were cultured in Dulbecco’s modified Eagle medium (DMEM, PAN Biotech, Wimborne, UK) (glucose concentration 4.5 g/L) supplemented with 10% fetal bovine serum (PAN Biotech, Wimborne, UK), 1 mM glutamax (Gibco, Invitrogen, Paisley, UK), 1 mM sodium pyruvate (Gibco, Invitrogen, Paisley, UK), and 50 μg/mL uridine (Sigma Aldrich, Lyon, France) at 37°C, 5% CO2. All cell lines were routinely tested for the absence of mycoplasma contamination.

### Methods details

#### Cell treatments

Drugs were added to culture medium at concentrations giving maximal inhibition of the targeted enzyme: 10 μM Carbonyl cyanide 3-chlorophenylhydrazone (CCCP, Sigma Aldrich), 0.6–1.4 μM Carbonyl cyanide 4-(trifluoromethoxy)phenylhydrazone (FCCP, Sigma Aldrich, Saint-Quentin-Fallavier, France) 2.5μM Rotenone (Sigma Aldrich), 2 μg/ml Antimycin A (Sigma Aldrich), 4 μg/mL Oligomycin (Sigma Aldrich), 30 μM BMS-199264 (Sigma Aldrich), 30 μM Carboxyatractyloside (Sigma Aldrich) (durations of treatment are indicated in the figure legends). All treatments and analyses were performed in cell culture medium, i.e., supplemented with 10% FBS in order to avoid stress-induced FBS deprivation.

#### Mitochondrial enzymatic activities

The specified activities of OXPHOS complexes were measured at 37°C on a UVmc2 spectrophotometer (SAFAS, Monaco) in a mitochondrial-enriched fraction from frozen cell pellets. The activities of NADH ubiquinone reductase (complex I, CI), ubiquinol cytochrome *c* reductase (complex III, CIII), F0F1-ATP hydrolase (complex V, CV) and citrate synthase (CS) were measured according to routine methods.^22^^,^^25^

#### Fluorescence microscopy and mitochondrial morphology

Fibroblasts were seeded in culture medium with 10% FBS on a two-well or four-well chamber coverslips (Ibidi, Gräfelfing, Germany) at least one day before imaging. Mitochondrial morphology was assessed by staining cells with 100 nM Mitotracker Green (Life Technologies, Carlsbad, CA, USA) for 30 min at 37°C. Mitochondrial membrane potential have be evaluated using 10 nM TMRM for 30 min at 37°C (Molecular Probes, CA, USA) within culture medium, containing or not the drugs.

Mitochondrial ATP was assessed using the BioTracker ATP-Red dye (MerckMillipore, USA)^26^ at 5 μM for 15 min at 37°C, as described.^27^ Fluorescence images were acquired with an inverted wide-field microscope ECLIPSE Ti-E (Nikon, Tokyo, Japan) equipped with a 100× oil immersion objective (Nikon Plan Apo100x, Tokyo, Japan, N.A. 1.45) and an Andor *NEO* sCOMS camera controlled by Metamorph 7.7 software (Molecular Devices, Sunnyvale, CA). A precision piezoelectric driver mounted underneath the objective lens allowed faster Z-step movements, keeping the sample immobile while shifting the objective lens. Thirty-five image planes were acquired along the z axis at 0.1 mm increments. Imaris 8.0 software (Bitplane, Zurich, Switzerland) was used for 3D processing and morphometric analyses. For mitochondrial network characterization, images were iteratively deconvolved using Huygens Essential software (Scientific Volume Imaging, Hilversum, The Netherlands). The cell volume and mitochondrial network were modeled in 3D, and thresholds were defined in order to classify mitochondria depending on their volume: <5 μm^3^ (fragmented), <20 μm^3^ (normal), >20 μm^3^ (connected). To characterize mitochondrial membrane potential and mitochondrial ATP, images were iteratively deconvoluted using Huygens Essential software (Scientific Volume Imaging, Hilversum, Netherlands). The cell volume and mitochondrial network were modeled in 3D, and thresholds were defined to classify mitochondria according to their mean fluorescence intensity.

#### Western blotting and blue native page

Cellular proteins were solubilized in a Laemmli buffer, and 40 μg were resolved by SDS-PAGE in 8% polyacrylamide gel. Proteins were transferred to a nitrocellulose membrane using a semi-dry transfer apparatus (iBlot 2 Dry blotting system, ThermoFisher, France). Mouse anti-OPA1 (BD Biosciences, 612607), and Mouse anti-SDHA ([2E3GC12FB2AE2], ab14715, Abcam) primary antibodies were used (dilution at 1:1000). Membranes were then incubated for 2 h in the dark, with anti-mouse and anti-rabbit coupled, respectively with Alexa Fluor 680 and Alexa Fluor 790 dyes (ab186694 and ab186697 Abcam, 1:10,000). Membranes were washed twice with TBS 1X–Tween 0.1% and once with TBS 1X. Fluorescence was detected at 700 and 800 nm with an Odyssey XF imaging system (LI-COR Biosciences, Bad Homburg, Germany). Band intensities were quantified with Image Studio software (LI-COR Biosciences, Bad Homburg, Germany).

For F0F1-ATP synthase assembly analyses, mitochondria were enriched by using differential centrifugation, as previously described^22^ incubated 10 min on ice with digitonin (4 mg/mL in PBS, v/v; 0.2% w/v). Then, digitonin was diluted by addition of cold PBS (5 v/v). Cells were centrifugated for 10 min at 10,000 g and 4°C to recover the mitochondria enriched fraction. The pellet was washed once in 1 mL cold PBS, centrifuged (10,000g, 4°C), resuspended at 10 mg/mL in AC/BT buffer (1.5M aminocaproic acid and 75 mM Bis–Tris/HCl, pH 7.0, supplemented with Complete Mini Protease Inhibitor (Roche Diagnostics, Stockholm, Sweden)) and frozen at −80°C until analysis. For Blue Native PAGE analyses, 30 μg of the samples were diluted at 2 mg/mL in AC/BT buffer and mitochondrial membrane proteins were solubilized by incubation with the dedicated detergent for 10 min at 4°C: (a) 3 g/g protein n-dodecyl β-*d*-maltoside for preparation of native F0F1-ATPsynthase and (b) 6 g/g protein digitonin (1.2% w/v) to detect the F0F1-ATPsynthase higher assembly forms. After centrifugation for 20 min, 20,000 g at 4°C, the supernatant was collected, and 5% Serva Blue G dye (Biorad, Marnes-la-Coquette, France) in 1 M aminocaproic acid/50 mM Bis–Tris/HCl, pH 7 was added (1/20 v/v), prior to loading. Dimer/monomer F0F1-ATP synthase were separated on Native PAGE Novex 3–12% Bis-Tris gels (Invitrogen) for 3 h and transferred on PVDF membranes (GE Healthcare, Velizy-Villacoublay, France) in cold blotting buffer (25 mM Tris, 192 mM glycine, pH 8.3, 20% methanol). Membranes were hybridized using dedicated monoclonal antibodies (ATPase Inhibitory Factor 1/IF1 (ab110277), ATP5b (ab14730), UQCRC2 (ab14745), SDHA (ab14715), Abcam). Anti-mouse and anti-rabbit peroxidase-linked secondary antibodies (GE Healthcare, Life Sciences) were used at 1/10,000 dilution and signals were detected using a chemiluminescence system (Super signal West Femto, Thermo Scientific, #34095) (Thermo Fisher Scientific, Waltham, USA). Acquisitions were performed using the Odyssey FC imaging system and analyzed using Image Studio Lite (LI-COR Biosciences, Lincoln, USA).

#### Mitochondrial respiration rates and mitochondrial membrane potential

Oxygraph-2k equipped with the two-channel O2k-Fluorescence LED2-Module (O2k-Fluorometer, Oroboros Instruments, Innsbruck, Austria) was used to simultaneously monitor both oxygen level and fluorescence. Measurements were performed under atmospheric pressure in the 2.0-mL incubation chambers kept at 37°C by Peltier control. Changes of oxygen concentration over time are monitored with polarographic oxygen sensors, simultaneously with the fluorescence signals monitored with the Fluorescence-Sensor LED2 module equipped with Green filter set for TMRM (excitation 525 nm/emission filter 580 nm) for both chambers.

Respiration rates on intact cells were measured in cell culture medium on 5-7.10^6^ cells. For the analysis of patient’s cells bioenergetics profiles, the analysis started with routine respiration measurement, which is defined as respiration in culture medium without additional substrates or effectors (cell endogenous respiration, corresponding to the cellular oxidative metabolism). Then, F0F1-ATP synthase was inhibited with oligomycin (4 μg/mL), allowing the measurement of non-phosphorylating respiration (Leak respiration) and corresponding mitochondrial membrane potential (ΔΨm). This non-phosphorylating respiration (O) was subtracted from Routine (R) one to calculate the cellular phosphorylating respiration (R-O). This was followed by progressive uncoupling of oxidative phosphorylation by stepwise titration of FCCP up to optimum concentrations (0.6–1.4 μM) allowing the measurement of the maximal endogenous respiration (cellular oxidative capacity) with maximal depolarization.

For the analysis of the inhibitor effect on mitochondrial respiration rate and (ΔΨm), the analysis started with routine respiration measurement, in absence of any additional inhibitor. After 3–5 min, Oligomycin (CV inhibitor, 4 μg/mL), Rotenone (CI inhibitor, 2.5 μM), Antimycin (CIII inhibitor, 2 μg/mL), combined Oligomycin/Antimycin, combined Oligomycin/Rotenone, or CCCP (10 μM) were added. Respiration rates and membranes potential were again measured during the 30 min incubation. Experiments using antimycin or rotenone alone ended by further F0F1-ATP synthase inhibition by Oligomycin. Finally, all experiments ended by CCCP (10 μM) addition to fully collapse the ΔΨm.

Respiration rates and ΔΨm on permeabilized cells were measured in respiratory buffer RB (10 mM KH2PO4, 300 mM mannitol, 10 mM KCl, 5 mM MgCl2, 0.5 mM EGTA and 1 mg/mL serum albumin bovine, pH 7.2). First, state 2 (non-phosphorylating) respiration and ΔΨm were measured after adding both the substrate of CI and CII, i.e., 2.5 mM pyruvate and 5 mM malate (CI substrates) and 10 mM succinate (CII substrate), allowing full TCA cycle operation. Then, submaximal ADP concentration (0.5 mM) was added to stimulate ATP synthesis. This ADP concentration allowed a phosphorylating respiration at ∼50- to ∼60% of maximal OXPHOS capacity close to the one observed on intact fibroblast cells.^22^ After 3 min stabilization, Oligomycin (4 μg/mL), Rotenone (2.5 μM), Antimycin (2 μg/mL), combined to Oligomycin or Antimycin or FCCP (1 μM) were added and respiration and ΔΨm again recorded. Finally, all experiments ended by FCCP addition to fully collapse the ΔΨm.

Mitochondrial membrane potential (ΔΨm) was measured simultaneously with oxygen consumption in both intact and permeabilized cells using 0.5 μM final of the membrane potential sensitive probe: TMRM (Tetramethylrhodamine methyl ester perchlorate) (Sigma Aldrich, Saint-Quentin-Fallavier, France), a cell-permeant positively charged dye that accumulates within mitochondrial according to the inside negative potential in energized mitochondria with concomitant change in fluorescence. At the TMRM concentration used, unquenching mode is operating: dye accumulating within energized mitochondria form aggregates thus quenching (decreasing) the fluorescence emission while subsequent depolarization results in release of the dye and unquenching of the loaded probe, increasing the fluorescence. These fluorescence changes are proportionally to the ΔΨm within the range of 3–7 millions cells. The O2K-Fluo LED2-module is operating with electric current as the primary signal. Stepwise additions of 0.125 μM TMRM were carefully performed to calibrate the raw fluorescence signal [V] and convert to μM TMRM, before and at the end of each experiment to ensure fluorescence conversion into nmol TMRM uptake and signal stability, respectively. TMRM uptake, Δ*Fluo*, as the semi-quantitative measurement of ΔΨm is calculated from delta nmoles TMRM, i.e., the delta between maximal fluorescence (complete ΔΨm collapsed and TMRM release) and the different energetic states analyzed.

#### Metabolites measurements

Cells were seeded on a 24-wells cell culture plate at 15 000 cells/well. One day after seeding, ethanol (1/2000) or dedicated inhibitors (Rotenone, Antimycin, Oligomycin or combination of Oligomycin/Antimycin) were added. Cellular glucose consumption and lactate production were determined on cell supernatant after 1 to 6 h of cell culture on an Atellica IM1600 apparatus using Glucose and Lactate assays for Atellica system (Siemens, Courbevoie, France).

### Quantification and statistical analysis

For all experiments, each control or patient fibroblast cell line was analyzed at least in duplicate (two biological replicates). Results are represented as mean *± SD*. The nonparametric Mann–Whitney test (∗ symbols) or Wilcoxon test for paired data (# symbols) were used for means comparison (Biostatgv: https://biostatgv.sentiweb.fr/, iPLesp UMR S 1136). All data were considered statistically significant at *p < 0.05*. For the comparison of each mutant fibroblasts to controls, the values of mutant cell lines that were beyond ±2 SD (standard deviation) compared to mean control cells were considered to be different.
