# Supplementary material for: Mitochondrial F0F1-ATP synthase governs the induction of mitochondrial fission
Source: iScience. 2024 Apr 24;27(5):109808. doi: 10.1016/j.isci.2024.109808 (PMC11089353; doi:10.1016/j.isci.2024.109808)
Supplement: Document S1. Figures S1–S8 and Table S1 [file mmc1.pdf]

iScience, Volume *27*

Supplemental information

Mitochondrial F0F1-ATP synthase governs the induction of mitochondrial fission

Charlène Lhuissier, Valérie Desquiret-Dumas, Anaïs Girona, Jennifer Alban, Justine Faure, Julien Cassereau, Philippe Codron, Guy Lenaers, Olivier R. Baris, Naïg Gueguen, and Arnaud Chevrollier

Supplemental information

Mitochondrial F0F1-ATP synthase governs the induction of mitochondrial fission

Charlène Lhuissier, Valérie Desquiret-Dumas, Anaïs Girona, Jennifer Alban, Justine Faure, Julien Cassereau, Philippe Codron, Guy Lenaers, Olivier R. Baris, Naïg Gueguen, Arnaud Chevrollier

patient	mutated gene	variant (cDNA)	Zygosity	sex	reference
Ctr, n = 7				5M, 2F	
NDUFV1	*NDUFV1*				
MTO1	*MTO1*				
ATP5O	*ATP5PO*	c.87+3A>G	homozygous	M	Ganapathi et al., 2022 [1]
TMEM70	*TMEM70*	c.317-2A>G	homozygous	F	

SI Table 1: Description of primary skin fibroblasts of healthy controls and patients with mitochondrial disease.

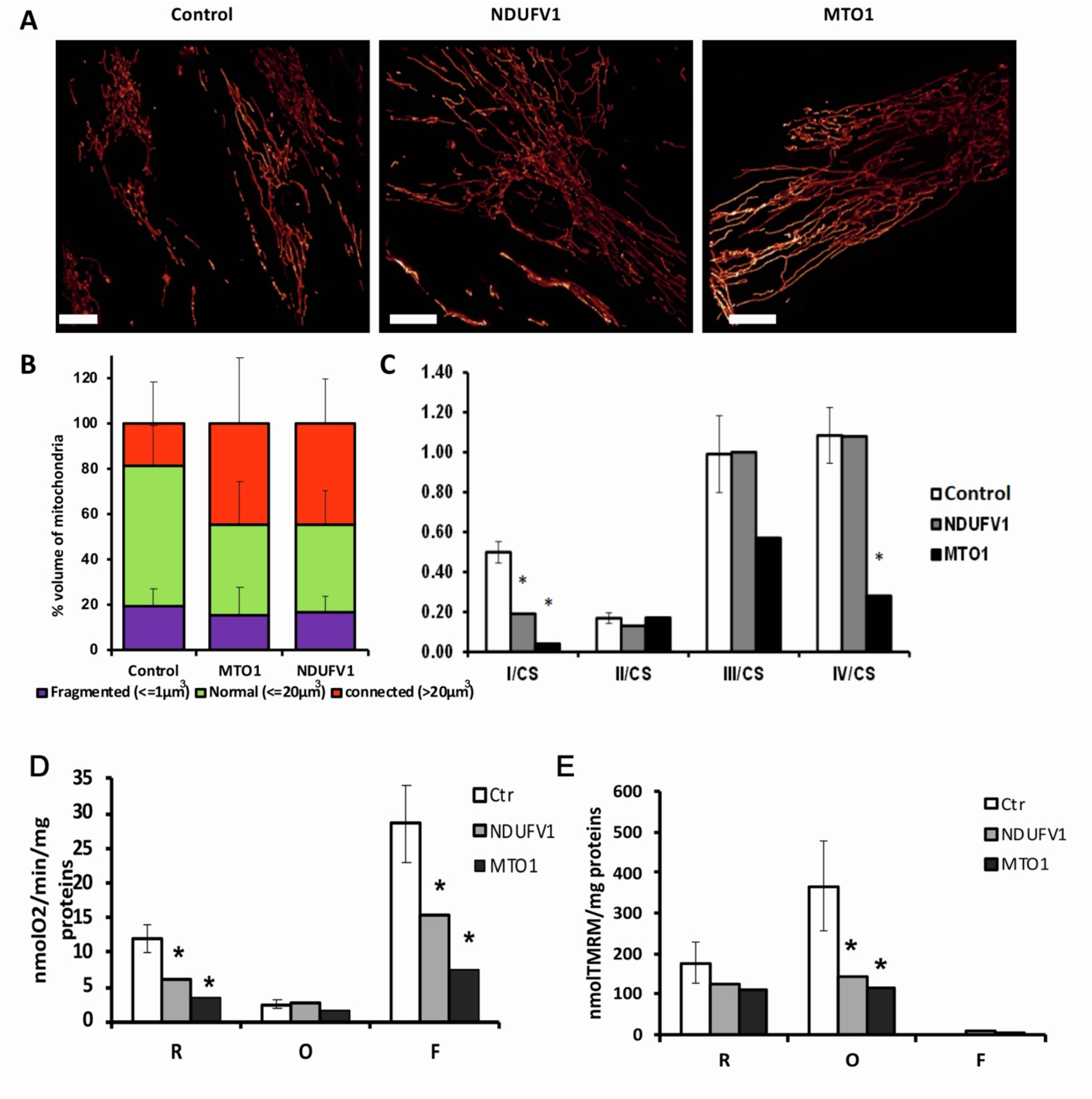

__Figure S1__: **The mitochondrial network displays a normal distribution in human patient skin fibroblasts.**

(A) Mitochondrial network observed with MitoTracker Green by fluorescence microscopy in patient fibroblasts with isolated CI deficiency (NDFUV1) or combined CI and CIV deficiencies (MTO1).

(B) **Quantification of mitochondrial volume.** The percentage of mitochondria volume represents the relative quantity of mitochondria. Controls: n=3, patient cells: analyses were performed on 12 images on triplicates. Results are presented as mean from each patient relative to control cells. In both mutated

cell lines, the mitochondrial network is not fragmented but rather hyperfused, despite their MRC defects.

(C) Enzymatic activities of the respiratory chain complexes, normalized to Citrate Synthase activity. Control, n=12. Each measurement was performed on two biological replicates in duplicate. Values are represented as a mean ±SD for control cells. * Indicate a significant difference (patient value beyond 2 SD to the mean, compared to control cells).

(D) Measurement of respiration rates in intact control and patient cells. Routine respiration measured corresponding to oxidative metabolism (R). Non-phosphorylating respiration rate (O), measured after inhibition of ATP synthesis through addition of Oligomycin (4 µg/ml). Maximal oxidative capacity (F), determined after uncoupling by FCCP.

(E) Mitochondrial membrane potential in intact patient cells. TMRM uptake was measured on control and patient intact cells in Routine condition and in non-phosphorylating condition after F0F1-ATP synthase inhibition by Oligomycin (O) in the same samples as in (D). TMRM uptake and respiration rates were normalized to cellular protein concentration. Controls, n=6, patients: analyses were performed on two independent biological replicates. Results are presented as means ± SD. * Indicates significant difference from Ctrl (value beyond 2 S.D. from controls).

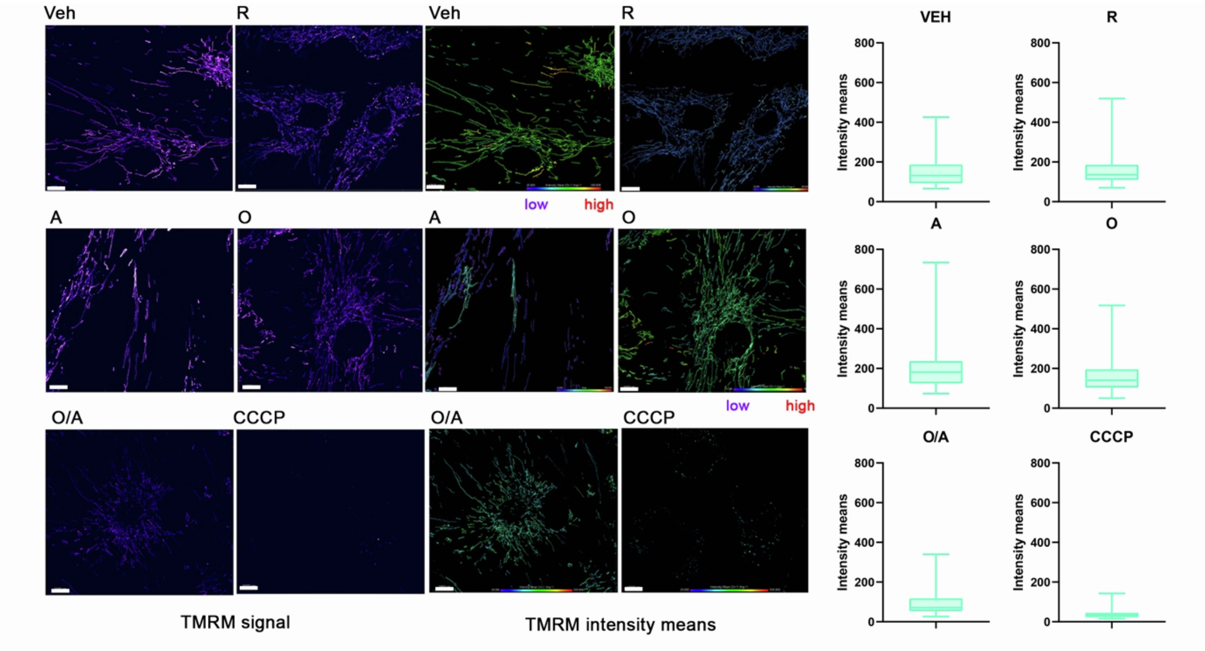

<u>Figure S2:</u> Inhibition of F0F1-ATP synthase does not decrease the mitochondrial membrane potential. Mitochondrial network observed with the TMRM probe by fluorescence microscopy in cells treated for 4h with Rotenone (R: 2.5µM), Antimycin (A: 2µg/ml), Oligomycin (O: 4µg/ml), Oligomycin (4µg/ml) combined to Antimycin (2µg/ml, O/A) and CCCP (10µM). The TMRM probe was added to the treatment medium and incubated for 30 minutes, without changing the medium, for the time of the acquisitions. n=4, analyses were performed on 6 images on four independent biological replicates. Results are presented as means ± SEM. * indicates significant difference from control conditions (Vehicle, Veh).

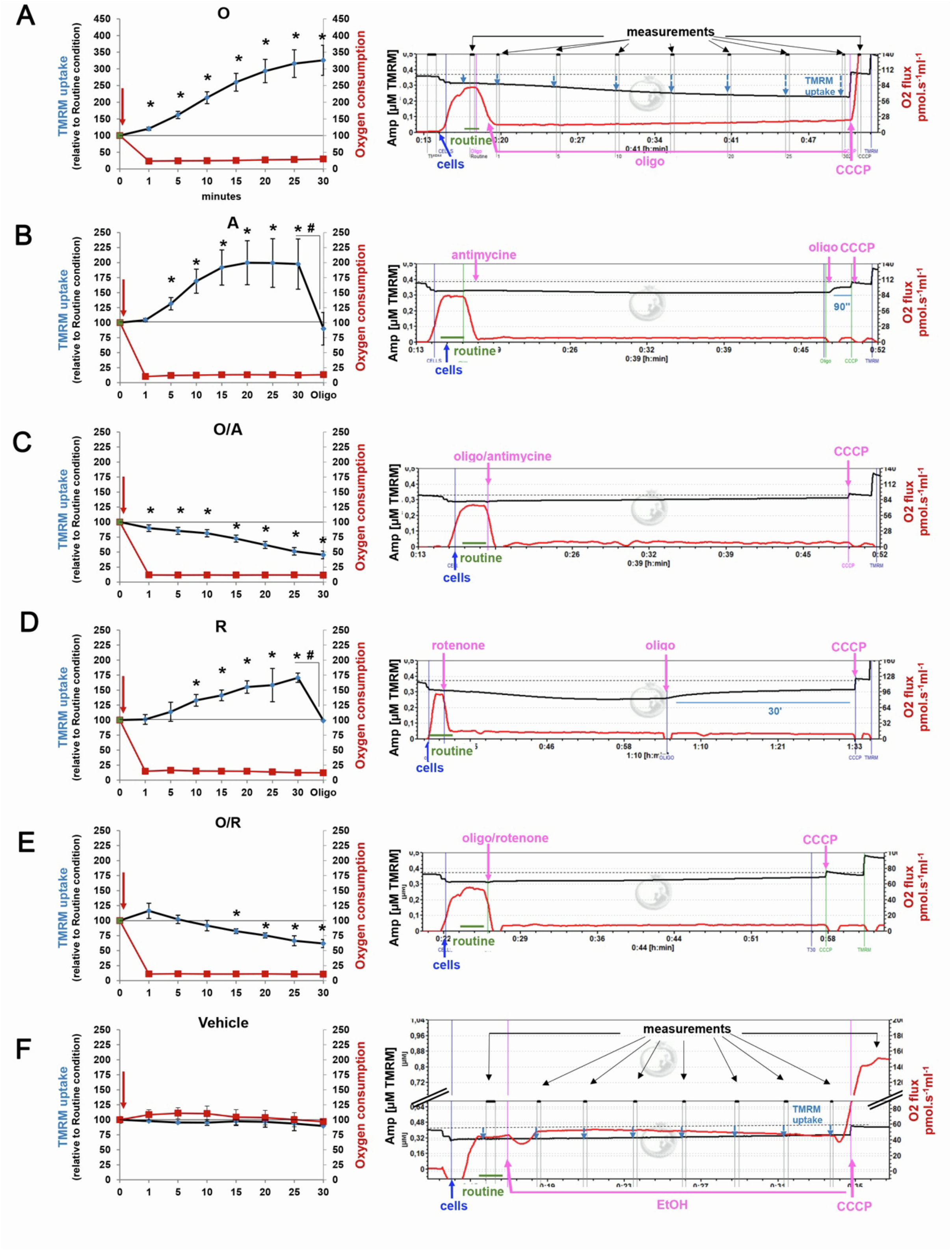

Figure S3: Modulation of the mitochondrial membrane potential and respiration rates over time with MRC or F0F1-ATPsynthase inhibitors. Intact cells were added to the oxygraphic chambers (Oroboros®) filled with culture medium and mitochondrial membrane potential (inversely proportional to TMRM fluorescence in this quenching mode, black trace) as well as the respiration rate (O2 flux, red

trace) were recorded. First, the Routine respiration, i.e. corresponding to the cellular oxidative metabolism and potential, were recorded for few minutes before inhibitors addition. Then, the dedicated inhibitor(s) (oligomycin: O, Antimycin: A, O/A, Rotenone: R or O/R) were added and both respiration and TMRM fluorescence were further recorded for 30 min. For A and R conditions, Oligomycin was further injected at the end of this period and depolarization checked. Finally, CCCP was used to fully depolarize mitochondria and checked for TMRM release. Left panel: quantitation of the time course evolution of respiration rates and mitochondrial TMRM uptake. Right panel: representative traces. Results are presented as means ± SEM.* indicates significant difference from Routine condition, # indicates significant effect of oligomycin addition relative to antimycin or rotenone.

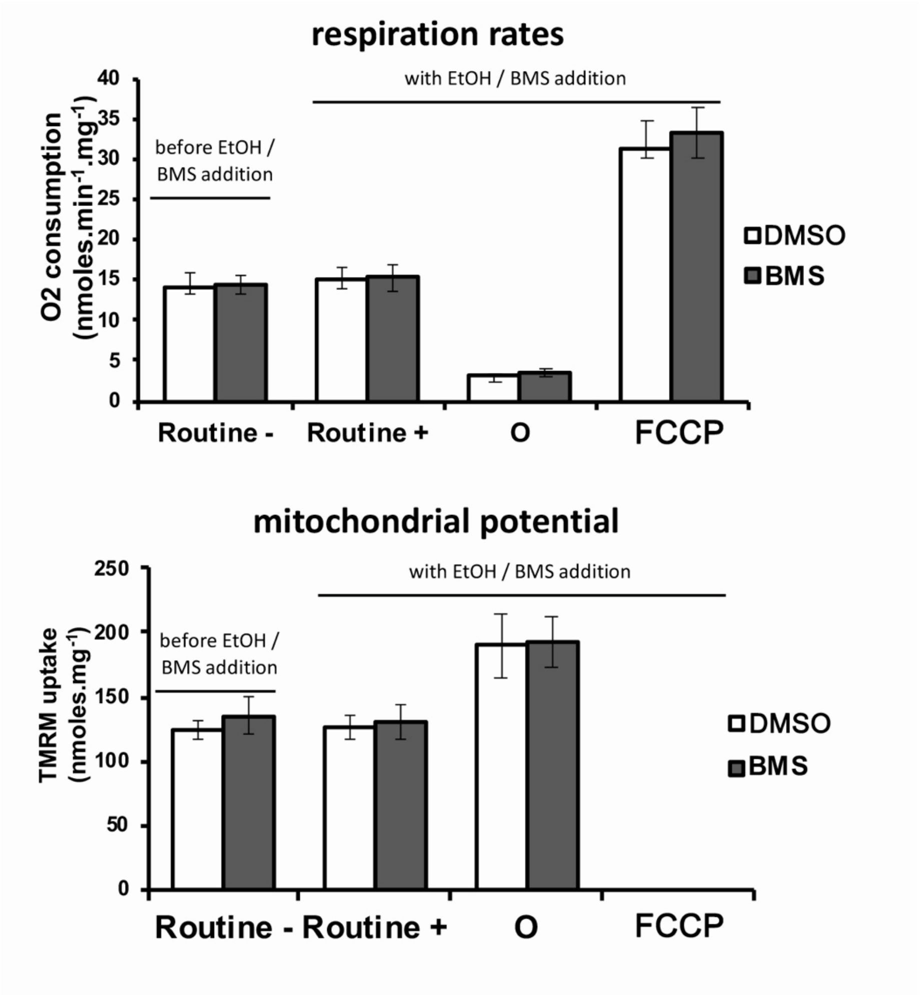

Figure S4: Inhibition of the hydrolase activity of F0F1-ATP synthase by BMS does not alter mitochondrial membrane potential or respiration rates.

(top) Measurement of respiration rates in vehicle and BMS-treated cells. Routine respiration measured on intact cells and corresponding to oxidative metabolism, before (Routine -) or after (Routine +) EtOH or BMS addition. Non-phosphorylating respiration rate (O), measured after inhibition of ATP synthesis through addition of Oligomycin (4 µg/ml). Maximal oxidation capacity (FCCP), determined after progressive uncoupling by FCCP titration (0.1 – 1.2 µM).

(**bottom**) **Mitochondrial membrane potential in vehicle and BMS-treated cells.** TMRM uptake measured in parallel on the same conditions (same samples). Results are presented as mean±S.E.M. (n=3).

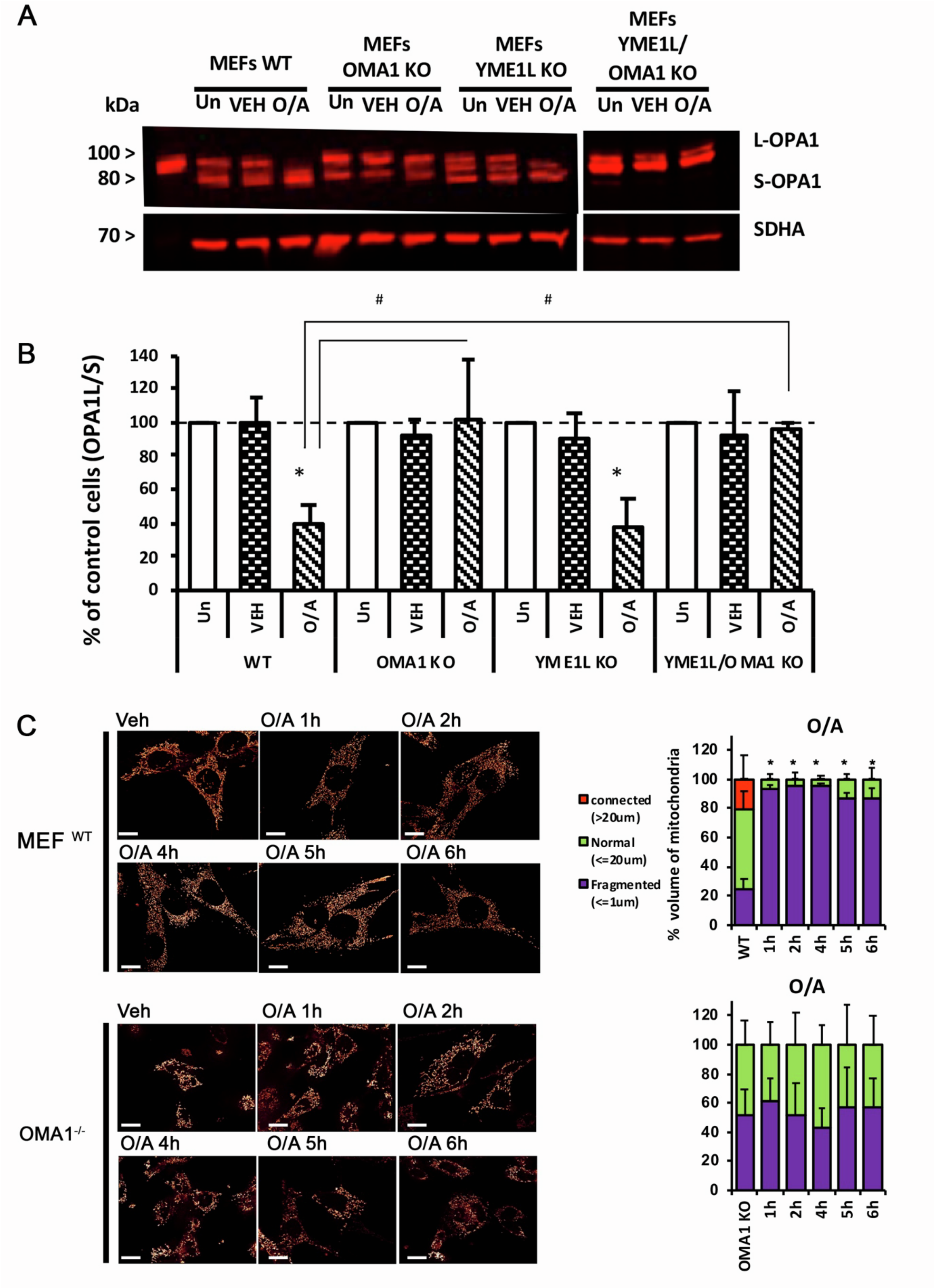

Figure S5: OPA1 protein is not cleaved in mouse embryonic fibroblasts MEFs KO for OMA1 protein. (A) OPA1 isoforms' expression profile: Representative images of Western-blots of OPA1

long (L) and short (S) isoforms and SDHA (mitochondrial loading reference) performed on protein extracts from treated-cells. **(B) Quantification of the OPA1 L/S ratio** relatively to each vehicle. n=4, in four independent biological replicates, each in duplicates. Results are presented as means ± SEM. * Indicates significant difference from control conditions (Veh). (**C) Mitochondrial network** observed with MitoTracker Green by fluorescence microscopy in MEFs wild type and OMA1-/-.

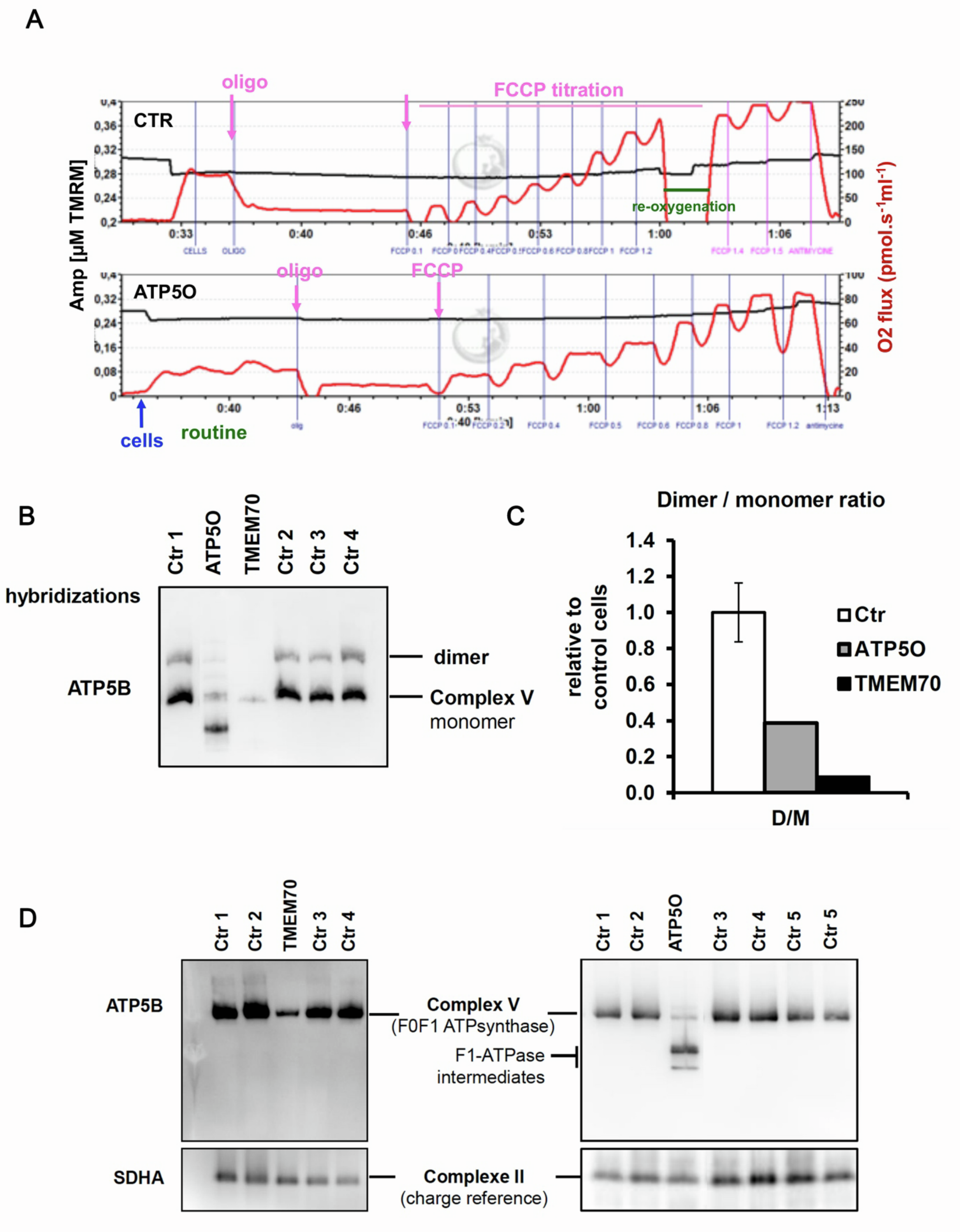

Figure S6: details of the analysis of the bioenergetic profile and F0F1-ATPsynthase assembly of ATP5O and TMEM70-mutated cells vs control cells. (A) The mitochondrial membrane potential

(inversely proportional to TMRM fluorescence, black trace) and respiration rates (O2 flux, red trace) of patient's cells were analyzed for Routine, non-phosphorylating and maximal, uncoupled conditions on intact cells. First, the Routine respiration, i.e. corresponding to the cellular oxidative metabolism, and potential were recorded after adding cells in the oxygraphic chambers. Then, oligomycin: O, was added to inhibit F0F1 ATP synthase and measure the non-phosphorylating respiration and potential. Finally, maximal oxidative capacity was measured by progressive uncoupling using FCCP until reaching the maximal respiration, with fully depolarize mitochondria. **(B, C, D)** Analysis of Complex V assembly in ATP5O and TMEM70 by Blue Native-PAGE electrophoresis and western blot. (B, C) : Supramolecular assembly of complex V into dimer vs monomer (digitonin-solubized mitochondrial extracts). D, E: Assembly of the CV holoenzyme (monomer, β-D-dodecylmaltoside-solubized mitochondrial extracts). Analyzed were performed in duplicate.

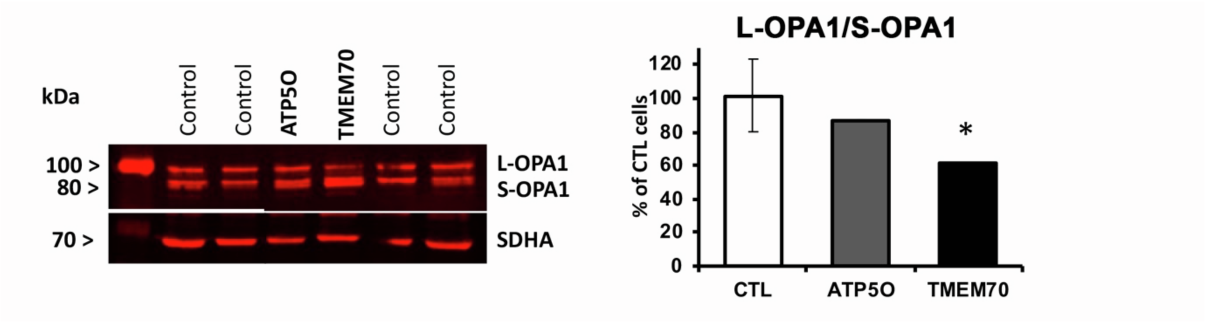

Figure S7: OPA1 processing in patient's cells.

OPA1 isoforms' expression profile in ATP5O and TMEM70 mutant cells: Left, Representative images of Western-blots of OPA1 long (L) and short (S) isoforms and SDHA (mitochondrial loading reference) performed on protein extracts from patient ATP5O and TMEM70 cells in left. Right panel, **Quantification of the OPA1 L/S ratio** relatively to the control cells. Control cells: n=4, analyses in two independent biological replicates, each in duplicates.

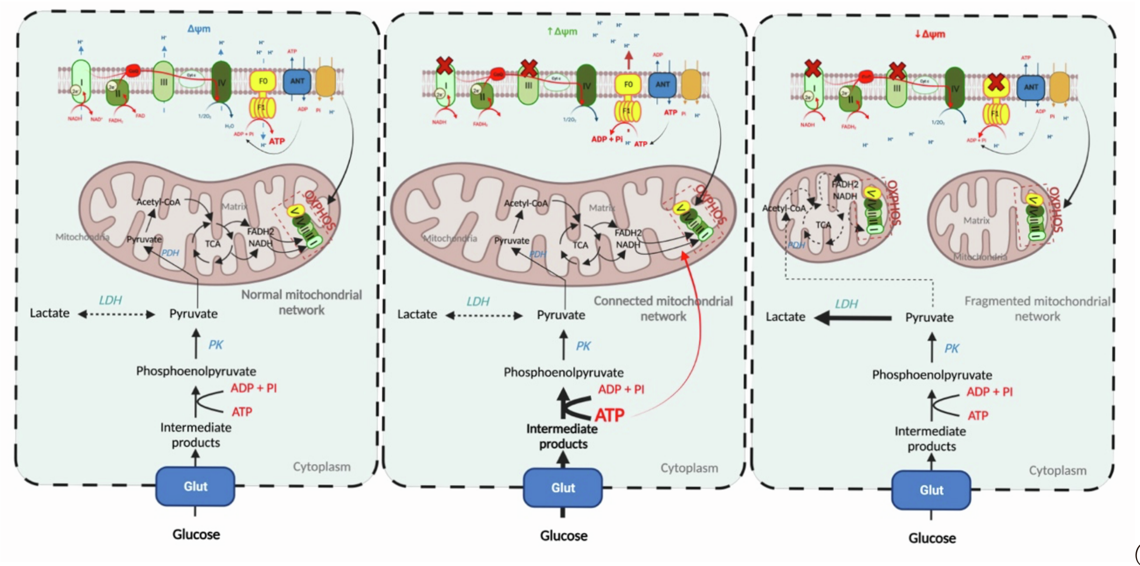

@

<u>Figure S8:</u> Mitochondrial adaptation prevent fission. Left : Normal condition; Center : MRC inhibition; Right: OXPHOS inhibition (MRC + F0F1-ATP synthase).

Reference :

1. Ganapathi, M., Friocourt, G., Gueguen, N., Friederich, M.W., Le Gac, G., Okur, V., Loaec, N., Ludwig, T., Ka, C., Tanji, K., et al. (2022). A homozygous splice variant in ATP5PO, disrupts mitochondrial complex V function and causes Leigh syndrome in two unrelated families. J Inherit Metab Dis *45*, 996-1012. 10.1002/jimd.12526.
